# Interfacial Microcompartmentalization by Kinetic Control of Selective Interfacial Accumulation

**DOI:** 10.1002/anie.202009701

**Published:** 2020-10-25

**Authors:** Qian Liu, Zhenyu Yuan, Meng Zhao, Max Huisman, Gido Drewes, Tomasz Piskorz, Serhii Mytnyk, Ger J. M. Koper, Eduardo Mendes, Jan H. van Esch

**Affiliations:** ^1^ Department of Chemical Engineering Delft University of Technology van der Maasweg 9 Delft 2629 HZ The Netherlands; ^2^ Department of Chemical Engineering East China University of Science and Technology Meilong 130 Shanghai 200237 P. R. China; ^3^ Department of Materials Science and Engineering Delft University of Technology Mekelweg 2 Delft 2628 CD The Netherlands

**Keywords:** interfaces, kinetics, microparticles, photochemistry, polymers

## Abstract

Reported here is a 2D, interfacial microcompartmentalization strategy governed by 3D phase separation. In aqueous polyethylene glycol (PEG) solutions doped with biotinylated polymers, the polymers spontaneously accumulate in the interfacial layer between the oil‐surfactant‐water interface and the adjacent polymer phase. In aqueous two‐phase systems, these polymers first accumulated in the interfacial layer separating two polymer solutions and then selectively migrated to the oil‐PEG interfacial layer. By using polymers with varying photopolymerizable groups and crosslinking rates, kinetic control and capture of spatial organisation in a variety of compartmentalized macroscopic structures, without the need of creating barrier layers, was achieved. This selective interfacial accumulation provides an extension of 3D phase separation towards synthetic compartmentalization, and is also relevant for understanding intracellular organisation.

## Graphical abstract

The phenomenon selective interfacial accumulation (SIA) is reported for an aqueous system. Based on interfacial migration, kinetic control and capture of the spatial organisation in a variety of compartmentalized macroscopic structures, without the need to create barrier layers between compartments, is possible. SIA opens an avenue for 3D phase separation approaches towards synthetic compartmentalized systems, and it is also relevant to understanding and mimicking intracellular organisation.

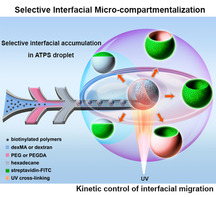

## Introduction

The spatial organisation of functional components in solution and at interfaces is essential for numerous biological and technical processes.^[1]^ In biology, intracellular organisation in organelles such as nucleus, mitochondria and Golgi apparatus enable otherwise incompatible processes in the confined space of the cell, whereas lateral membrane organisation like lipid rafts plays an important role in recognition and transport.^[2]^ In technology, for instance the controlled delivery of active ingredients, microreactors, and multicomponent sensing arrays all depend on the ability to organise the active components with different functions in space.^[3]^

Nowadays, synthetic bottom‐up approaches like molecular self‐assembly, phase separation and covalent entrapment towards the spatial organisation of 3D phases are well established,^[4]^ and numerous reports deal with the encapsulation and compartmentalization of bulk phases via membrane formation,^[5]^ micelles and supramolecular capsules,^[6]^ metal‐ or covalent organic frameworks,^[7]^ or phase separated systems of immiscible polymers.^[8]^ In sharp contrast, bottom‐up synthetic approaches to spatial organisation at interfaces are limited to phase separation of block copolymers,^[9]^ surfactants and surface adhesive molecules^[10]^ into distinct microdomains in thin polymer films, surfactant mono‐ and bilayers, and self‐assembled layers, respectively, and always involve the formation of at best a semi‐permeable layer at a solid–liquid or liquid‐liquid interface. Moreover, in most of the synthetic approaches interfacial and bulk spatial organisation remain disconnected, while in biological systems intracellular and lateral membrane organisation are intimately connected to enable signal transduction and cellular response.^[11]^ Therefore, the development of novel microcompartmentalization approaches connecting 2D and 3D spatial organisation is highly relevant for materials science and bioengineering.

**Scheme 1:**
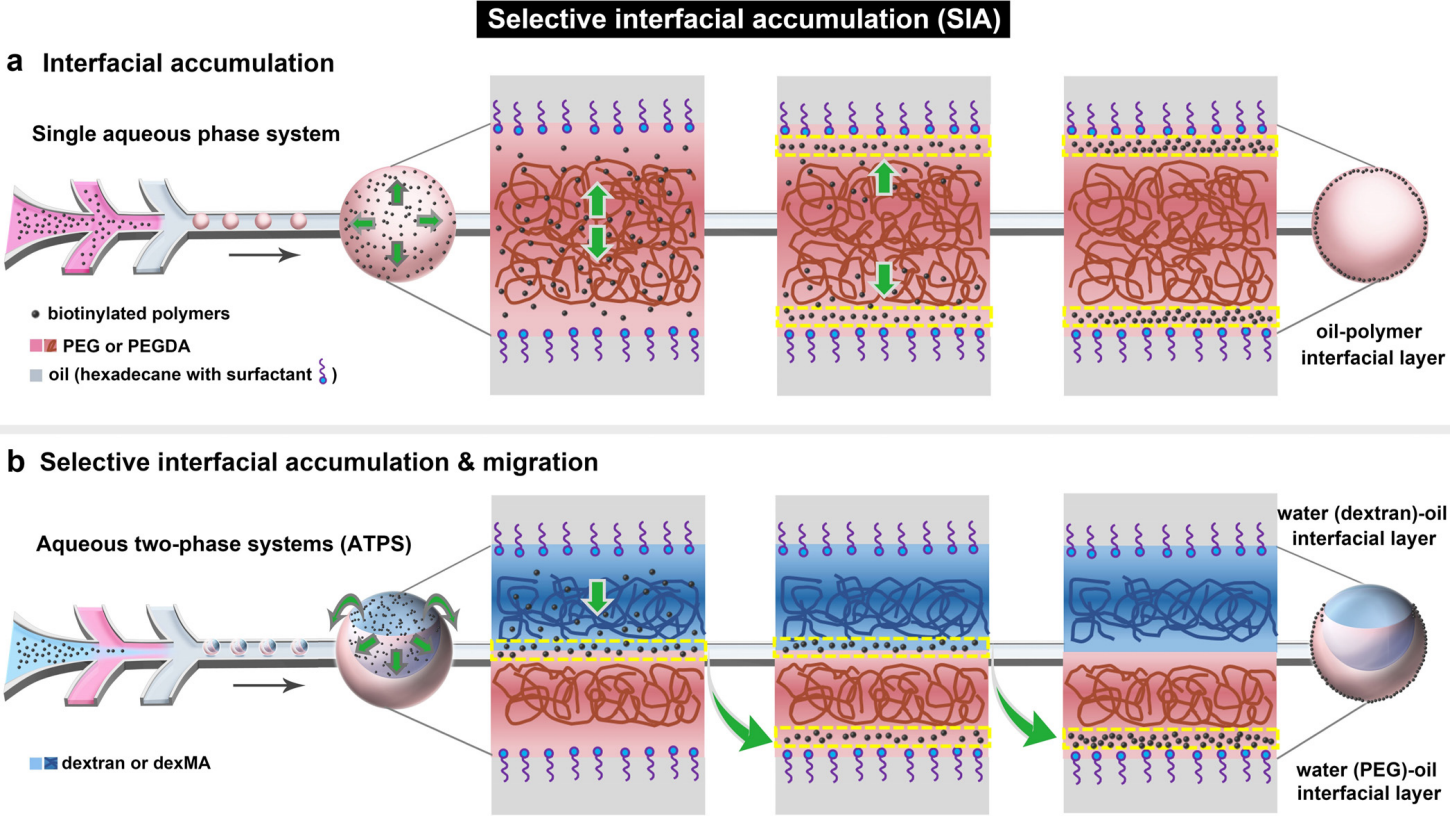
Interfacial microcompartmentalization by selective interfacial accumulation (SIA). a) Schematic of the migration and interfacial accumulation of biotinylated polymers (black dots) in PEG (brown lines) droplets which were produced in the microfluidic device. b) Schematic of the selective interfacial accumulation of biotinylated polymers in ATPS droplet of dextran (blue lines) and PEG (brown lines), and their interfacial migration from the water‐water interfacial layer to the water (PEG)‐oil interfacial layer.

Here, we report a novel interfacial microcompartmentalization approach in aqueous systems that is governed by 3D phase separation. We serendipitously found that in crowded aqueous solutions of polyethylene glycol (PEG) polymers doped with biotinylated polymers, the biotinylated polymers spontaneously migrate to and accumulate in the oil‐polymer interfacial layer between the oil‐surfactant‐water interface and the adjacent PEG polymer phase (Scheme 1 a). By extending the system to an aqueous two‐phase systems (ATPS) of PEG and dextran, as shown in Scheme 1 b, we found that the biotinylated polymers first accumulate in the water‐water interfacial layer between the PEG and dextran phase, and then selectively migrate along this interfacial layer to the water (PEG)‐oil interfacial layer, but never to the water (dextran)‐oil interfacial layer. By equipping the different polymers with photopolymerizable groups, we have been able to kinetically control and capture the spatial organisation in a variety of compartmentalized macroscopic structures, without the need to create barrier layers between compartments. Therefore, we strongly believe that this new phenomenon coined as “selective interfacial accumulation (SIA)” provides an highly significant extension of current 3D phase separation approaches towards synthetic compartmentalized systems, and is also highly relevant to the understanding and mimicking of interlinked membrane and intracellular organisation in living systems.

## Results and Discussion

### Selective Interfacial Accumulation (SIA)

Hydrogel microparticles were functionalized with biotinylated molecules and produced in a microfluidic device. As shown in Video 1 and Figure S1 (see the Supporting Information), biotin‐PEG‐thiol (biotin‐PEG‐SH, M_W_=788) was, respectively mixed with a photo‐crosslinkable polymer solution of dextran methacrylate (dexMA) and polyethylene glycol diacrylate (PEGDA). Microdroplets were produced in the microfluidic device by injecting this polymer aqueous solution into the oil phase (hexadecane) with a production rate of ≈180 droplets per minute and cross‐linked by UV irradiation. During the cross‐linking process, biotin‐PEG‐SH was covalently bound in the hydrogel network by thiol‐ene click reaction.^[12]^ After collecting and washing the particles, the location of biotin‐PEG‐SH in the hydrogel microparticles was probed by binding of streptavidin‐FITC based on the strong biotin‐streptavidin noncovalent interaction,^[13]^ and imaged by confocal laser scanning microscopy (CLSM).

**Figure 1:**
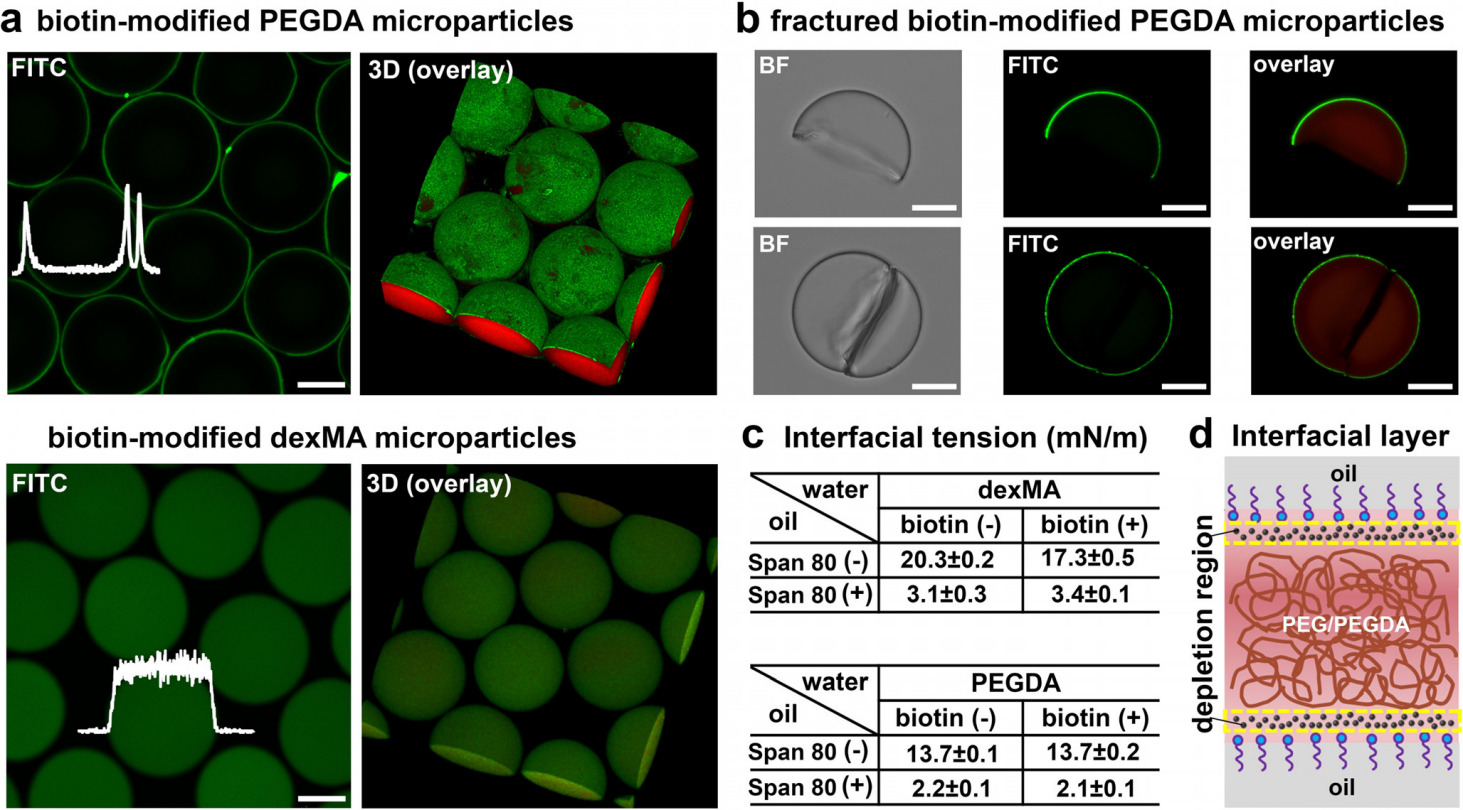
Selective interfacial accumulation (SIA). a) CLSM and 3D images of the biotin‐modified PEGDA microparticles and the biotin‐modified dexMA microparticles after reacting with streptavidin‐FITC. The fluorescence intensities of FITC (white) were inserted. The concentration of biotin‐PEG‐SH was 4 mg mL^−1^. PEGDA and dexMA hydrogel were labelled by methacryloxyethyl thiocarbamoyl rhodamine B (red). b) Bright‐field (BF) and CLSM images of fractured biotin‐modified PEGDA microparticles after reacting with streptavidin‐FITC. c) Interfacial tension of dexMA (25 % w/w) and PEGDA (28.6 % w/w) droplets in oil phase in the absence (−) and presence (+) of biotin‐PEG‐SH (4 mg mL^−1^). Hexadecane without (−) and with (+) surfactant (Span 80, 3 % w/w) were used as the oil phase. d) Schematic of the accumulation of biotin‐PEG‐SH in the water (PEG/PEGDA)‐oil interfacial layer. The black dots represent biotin‐PEG‐SH. Scale bar 50 μm.

Unexpected results were obtained for these biotin‐modified hydrogel microparticles. Streptavidin‐FITC diffused into the hydrogel, but did not bind to the unmodified microgels (Figure S2). However, as shown in Figure 1 a, the biotin‐modified dexMA microparticles give a homogeneous fluorescent signal throughout the particle interior, while the green fluorescence was confined to the surface of the biotin‐modified PEGDA hydrogel microparticles. Such observed exclusive binding of streptavidin‐FITC to the PEGDA particles surface could be explained by one of the two potential pathways that we envisioned. In the first scenario, biotin‐PEG‐SH exists homogeneously in the particles, but binding of streptavidin‐FITC leads to the formation of a barrier layer, which prevents further diffusion of streptavidin‐FITC into the PEGDA hydrogel network interior, thus resulting in a heavily modified outer layer and no streptavidin‐FITC within the bulk of the particles. In the second case, biotin‐PEG‐SH is exclusively immobilized on the surface of the PEGDA gel particles and the signal of streptavidin‐FITC from the CLSM images properly reflects the location of biotin‐PEG‐SH in these particles.

To investigate the possible formation of a surface barrier layer binding of streptavidin‐FITC on the PEGDA hydrogel microparticles, we fractured the biotin‐modified PEGDA gel particles by exposing them to ultrasound (around 30 min), after which they were reacted with streptavidin‐FITC. As shown in Figure 1 b, after washing with water, fluorescence was only observed on the surface of the PEGDA microparticles, while fluorescence was neither observed on the cross‐sectional fracture surface, nor inside the hydrogel. This result implies that binding of streptavidin does not lead to a barrier layer at the microparticles surface, while it confirms the absence of biotin‐PEG‐SH in the PEGDA particle interior. Therefore, we conclude that streptavidin‐FITC reflects the location of biotin‐PEG‐SH in the microparticles, and biotin‐PEG‐SH is homogeneously present in the dexMA hydrogel particles interior, but only present at the surface of the PEGDA hydrogel particles.

To explore why biotin‐PEG‐SH molecules selectively compartmentalized at the surface/interface of the PEGDA hydrogel microparticles, the biotin‐PEG‐SH was hypothesized as a surfactant and driven to the water (PEGDA)‐oil interface by a decrease of interfacial free energy. So we measured the interfacial tension of the dexMA and PEGDA droplets with and without the biotin‐PEG‐SH. As shown in Figure 1 c, no significant reduction of the interfacial tension was observed after adding biotin‐PEG‐SH in both dexMA and PEGDA, neither with nor without surfactant (Span 80) in oil. These results clearly indicate that biotin‐PEG‐SH is not surface/interface active and does not lead to a reduction of the interfacial energy of the water (PEGDA)‐oil interface. An alternative explanation for the exclusive location of biotin‐PEG‐SH in the PEGDA microparticles comes from the widespread notion that with interface in contact with polymer solutions, like the water‐oil interface, a thin interfacial layer is depleted from the polymer due to the fact that the polymer repels the solvent.^[14]^ Most likely, the biotin‐PEG‐SH accumulated in this depletion region between the oil‐surfactant‐water interface and the adjacent PEGDA phase (Figure 1 d), probably driven by osmotic forces^[15]^ and/or unfavourable interaction with the PEGDA phase (see supporting Langmuir‐McLean equation), which coined a novel phenomenon, selective interfacial accumulation (SIA). So far, to the best of our knowledge, this SIA has not been reported in soft materials, but it may have some resemblance to the grain boundary segregation in materials science.^[16]^ As it spontaneously forms interfacial micro‐compartments in a high concentration polymer aqueous solution, it also may be highly relevant to specific microphase separation, in particular intracellular spatial organisation.

### Kinetic Control of the Selective Interfacial Accumulation (SIA)

To further explore the SIA and achieve diversities of interfacial microcompartmentalization, the biotin‐PEG‐SH was introduced into aqueous two‐phase systems (ATPS) of PEG/PEGDA and dextran/dexMA. Biotin‐PEG‐SH was only added into one of the two aqueous phases, following by crosslinking of the acrylate/methacrylate modified phase (PEGDA or dexMA) and removal of the uncrosslinked phase (dextran or PEG),^[17]^ to, respectively yield crescent‐shaped PEGDA hydrogel microparticles and ellipsoidal dexMA hydrogel microparticles (Video 1, Figures S3 and S4). It should be noted that the inner surface of the crescent‐shaped particles and the curved side of the ellipsoidal particles correspond to the water‐water interface, the outer surface of the crescent‐shaped particles corresponds to the water (PEG/PEGDA)‐oil interface, and the flat side of the ellipsoidal particles is relevant to the water (dextran/dexMA)‐oil interface (Figure S5). Experiments with the unmodified crescent‐shaped and ellipsoidal microparticles were demonstrated in Figures S6 and S7. The location of biotin‐PEG‐SH in the ATPS droplets was captured by fixing it in the hydrogel network at different cross‐linking rates by varying the concentration of photo initiator (LAP).^[18]^

**Figure 2:**
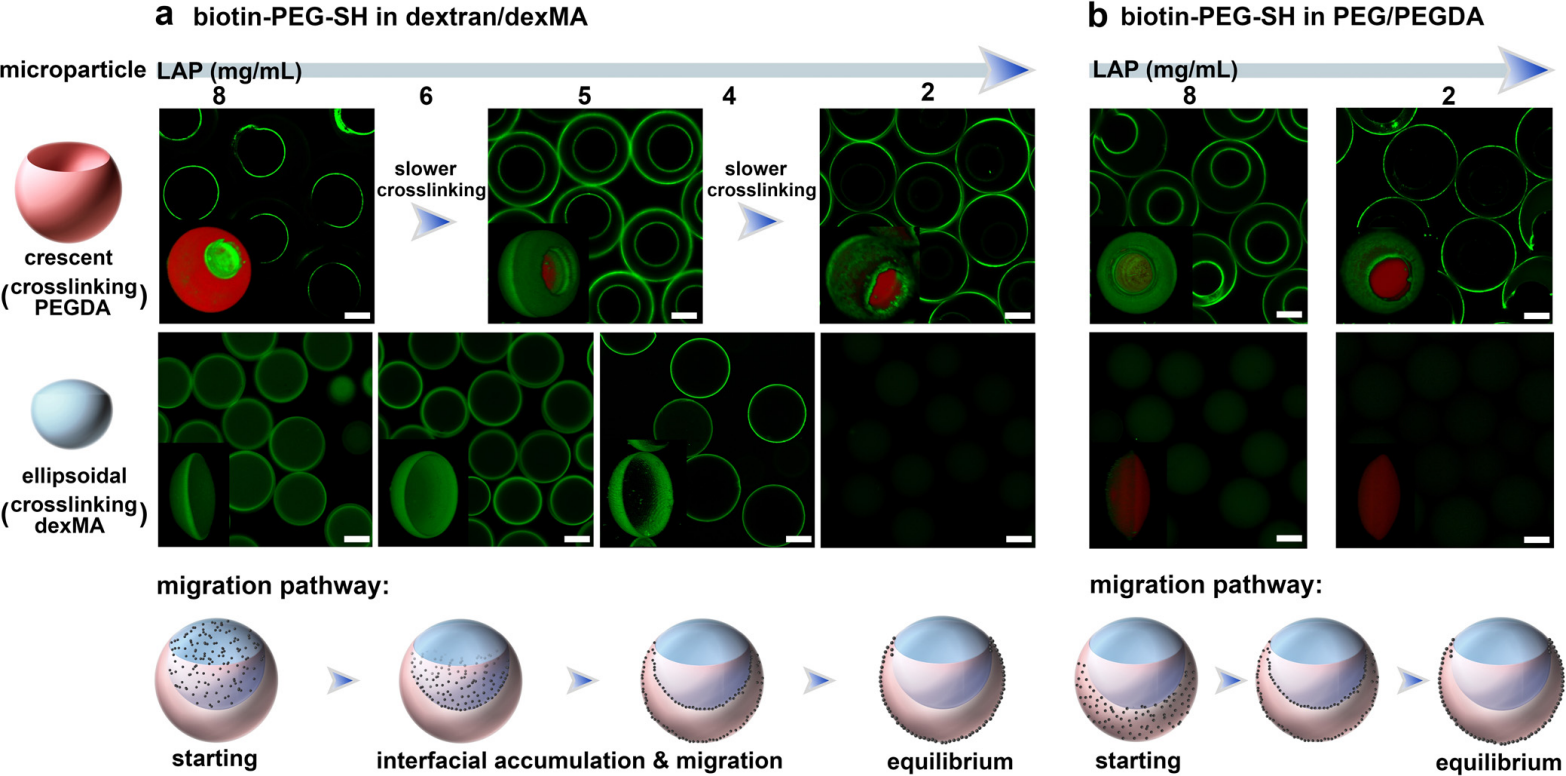
Kinetic control of the selective interfacial accumulation (SIA) in ATPS. a) When biotin‐PEG‐SH (4 mg mL^−1^) was added in dextran/dexMA, CLSM images of the crescent‐shaped PEGDA (top, merged signals) and ellipsoidal dexMA (bottom, FITC) microparticles created by varying the concentration of LAP (from 8 to 2 mg mL^−1^), and the corresponding schematic of migration pathway of biotin‐PEG‐SH in the ATPS droplet. b) When biotin‐PEG‐SH was added into PEG/PEGDA, CLSM images of the crescent‐shaped PEGDA (top, merged signals) and ellipsoidal dexMA (bottom, merged signals) microparticles crosslinked at 8 and 2 mg mL^−1^ of LAP (from left to right), and the corresponding schematic of migration pathway. 3D images were inserted. The PEGDA and dexMA microparticles were crosslinked with methacryloxyethyl thiocarbamoyl rhodamine B (red), then reacted with streptavidin‐FITC (green). Scale bar 50 μm.

Firstly, biotin‐PEG‐SH was added in the dextran phase and the PEGDA phase was crosslinked at different rates to form the crescent‐shaped microparticles. As shown in Figure 2 a up panel and Figure S9, when the PEGDA was crosslinked fast (LAP=8 mg mL^−1^), biotin‐PEG‐SH was present only on the inner surface of the crescent‐shaped particles, while decreasing the LAP concentration to 5 mg mL^−1^ leads to a biotinylated compartment on both inner and outer surfaces of the particles. When the gel particles were crosslinked at an even lower concentration of LAP (2 mg mL^−1^), the biotinylated compartment was located only on the outer surface of the crescent‐shaped particles. Meanwhile, different interfacial compartmentalization was obtained by crosslinking the droplet produced in microfluidic channels with different length leading to different residence times (Figure S10), indicating the interfacial compartmentalization occurs before polymerization. Most remarkably, in each of the above‐mentioned experiments, no matter under which condition of crosslinking, biotin‐PEG‐SH was only observed on the microparticles surface and no streptavidin‐FITC signal was observed inside the PEGDA hydrogel network. Apparently, after adding biotin‐PEG‐SH polymers into the dextran phase (Scheme 1 b), they diffuse from the dextran phase to the water‐water interfacial layer which causes the inner surface compartmentalization on the crescent‐shaped microparticles, and then continue to migrate along this interfacial layer to the water (PEGDA)‐oil interfacial layer leading to more different types of interfacial microcompartmentalization, and the migration process does not go through the PEGDA bulk polymer phase. Similar results were obtained by varying the volume ratio between PEGDA and dextran phases (Figure S12), and also lead to exclusive interfacial compartmentalization.

Secondly, biotin‐PEG‐SH was added in the dexMA phase, following by crosslinking of the dexMA phase at different rates to yield various ellipsoidal microparticles. As shown in Figure 2 a bottom panel and Figure S13, at high cross‐linking rates using 8 mg mL^−1^ of LAP, ellipsoidal microparticles were obtained with the modification of biotin‐PEG‐SH inside the gel network and on the curved side. At slower crosslinking rates, the streptavidin‐FITC signal has disappeared from the hydrogel interior, and a biotin‐compartment appeared exclusively on the curved side of the ellipsoidal particles first as a shell (6 mg mL^−1^ LAP) and at a later stage as a ring (4 mg mL^−1^ LAP). However, the biotin‐compartment never appeared on the flat side of ellipsoidal dexMA particles corresponding to the water (dexMA)‐oil interface. When the concentration of LAP was decreased to around 2 mg mL^−1^, neither the interior nor the curved and flat surfaces of the ellipsoidal particles displayed any FITC signal, indicating that all biotin‐PEG‐SH has diffused out of the dexMA phase and its interfacial layers.

Altogether, from these combined observations the preferred migration pathway of biotin‐PEG‐SH in the ATPS droplet becomes clear: when biotin‐PEG‐SH is initially present in the dextran/dexMA phase, it first diffuses towards the PEG/PEGDA phase but does not enter the PEG/PEGDA phase. Instead, biotin‐PEG‐SH compartmentalizes at the water‐water interfacial layer between the two polymer phases and from here on continues to migrate along this interfacial layer to the PEG/PEGDA adjacent water‐oil interfacial layer, and reaches the equilibrium at this interfacial layer (schematic of migration pathway in Figure 2 a and Scheme 1 b). It also indicates that the polymerization of PEGDA and dexMA only capture the intermediate state of biotinylated polymers during their migration, but their relative rates are not relevant to induce the selective interfacial compartmentalization.

Furthermore, we have examined the migration pathway of biotin‐PEG‐SH when it is initially present in the PEGDA (or PEG) phase. As shown in Figure 2 b and Figure S14, fast cross‐linking of PEGDA phase (8 mg mL^−1^ LAP) leads to the biotinylated compartment appeared on both inner and outer surfaces of the crescent‐shaped microparticles, while cross‐linking of the dexMA phase yields a small biotin‐compartment on the curved side of ellipsoidal particles. Apparently, biotin‐PEG‐SH has diffused out of the PEGDA phase and migrated to the interfacial layers before cross‐linking could take place, but did not yet reach an equilibrium distribution presumably due to the fast crosslinking. When the crosslinking was slowed down by decreasing LAP concentration to 2 mg mL^−1^, the biotin‐compartment was only present on the outer surface of crescent‐shaped particles, while no biotin‐PEG‐SH was observed in the ellipsoidal particles. These results indicate that when biotin‐PEG‐SH is initially present in the PEG/PEGDA phase, it diffuses rapidly from the PEGDA phase to the nearest PEG/PEGDA adjacent interfacial layers but does not diffuse into the dexMA phase. Instead, the biotin‐PEG‐SH continues to migrate along the interfacial layer to finally accumulate in the water (PEG/PEGDA)‐oil interfacial layer (schematic of migration pathway in Figure 2 b).

Overall, the results from these experiments confirm the selective interfacial accumulation (SIA) of biotin‐PEG‐SH in the PEG/PEGDA adjacent interfacial layers (as shown in Scheme 1 b): the water‐water and the water (PEG/PEGDA)‐oil interfacial layers in the ATPS droplets. Moreover, by kinetic arrest of the interfacial migration of biotin‐PEG‐SH at different crosslinking rates, we have been able to selectively tailor a biotin‐functionalized microcompartment which is a universal adapter to most biofunctional molecules, at different surfaces/interfaces of the crescent‐shaped and ellipsoidal hydrogel microparticles during their formation.

### Manipulation of the Selective Interfacial Accumulation (SIA)

**Figure 3:**
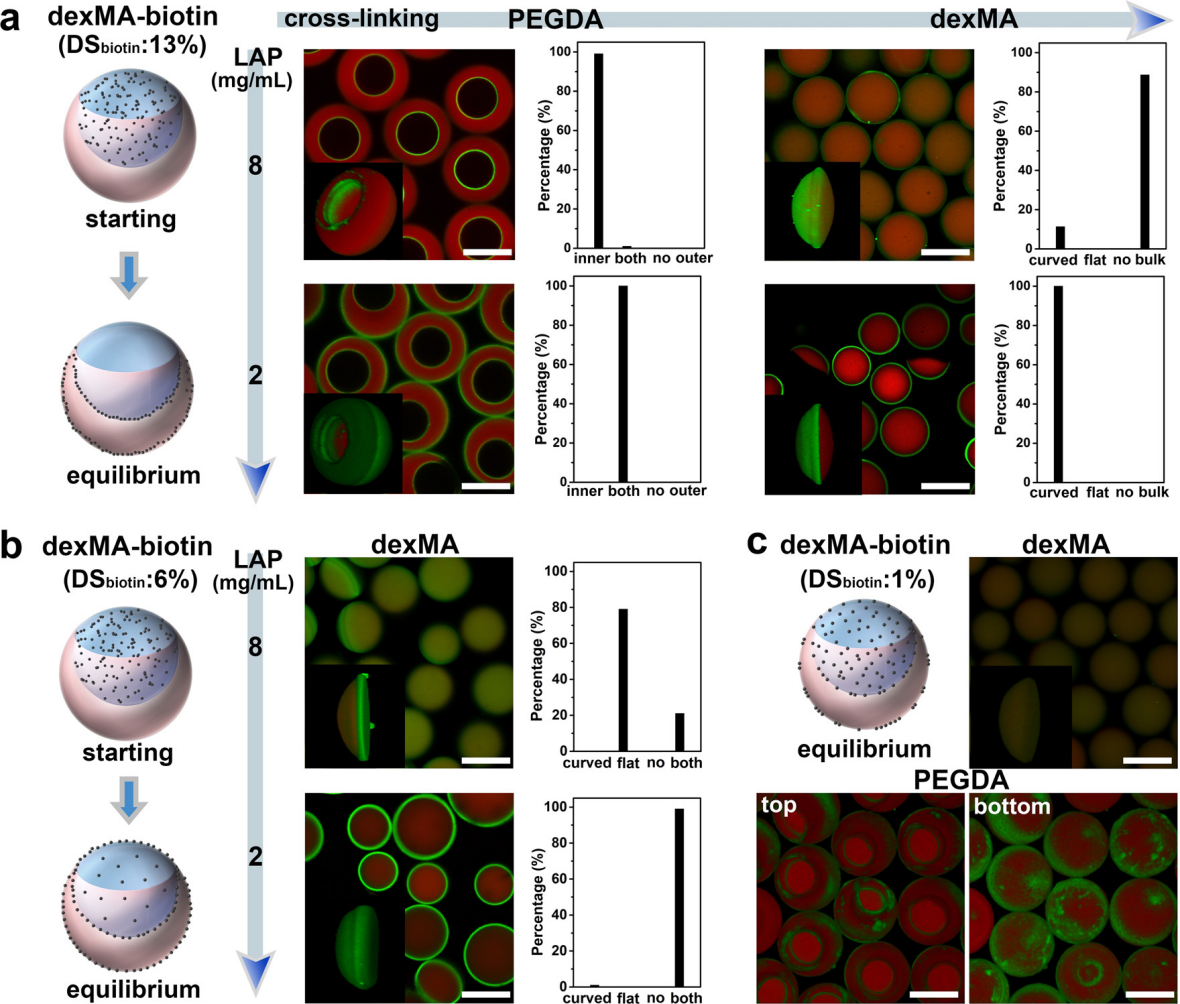
Manipulation of the selective interfacial accumulation (SIA) in ATPS by varying the polymer structure and DS_biotin_. a) When dexMA‐biotin (DS_biotin_=13 %) was added in dexMA/dextran, CLSM images of the crescent‐shaped and ellipsoidal microparticles crosslinked at 8 (up) and 2 (down) mg mL^−1^ of LAP, and their corresponding statistics of biotin‐compartment distribution. b) When dexMA‐biotin (DS_biotin_=6 %) was added in dexMA, CLSM images of the ellipsoidal dexMA microparticles crosslinked at 8 (modification on the flat side) and 2 (modification on both sides) mg mL^−1^ of LAP, as well as their statistics of biotin‐compartment distribution. c) When dexMA‐biotin (DS_biotin_=1 %) was added in dexMA/dextran, CLSM images of ellipsoidal dexMA particles and 3D CLSM images of patchy crescent‐shaped PEGDA particles (2 mg mL^−1^ LAP) from top and bottom view. Every statistic was taken by at least 100 particles. DexMA‐biotin was 4 mg mL^−1^. Scale bar 100 μm.

To further investigate the factors affecting the SIA and manipulate the microcompartmentalization of biotinylated polymers at different interfacial layers in the ATPS, the polymer skeleton structure was changed from PEG to dextran, and the reactive group was changed from thiol to methacrylate. Biotin decorated dexMA (dexMA‐biotin, M_W_=20 000) with different degrees of biotin substitution (DS_biotin_) was synthesised (Figures S15 and S16). No significant change of the interfacial tension was observed after adding dexMA‐biotin (Table S1). As shown in Figure 3 a, dexMA‐biotin (DS_biotin_ of 13 %) was added into the dextran/dexMA. Under the condition of fast crosslinking PEGDA (LAP=8 mg mL^−1^), dexMA‐biotin was compartmentalized at the inner surface of crescent‐shaped microparticles. When the crosslinking rate was slowed down (LAP=2 mg mL^−1^), the compartment of dexMA‐biotin was on both inner and outer surfaces of the crescent‐shaped microparticles. The interfacial compartmentalization implies that the thiol‐ene reaction is not relevant to induce the SIA. When the dexMA was crosslinked at high (LAP=8 mg mL^−1^) rate, dexMA‐biotin was present in the interior hydrogel. While at low crosslinking rate (LAP=2 mg mL^−1^), the dexMA‐biotin was compartmentalized on the curved side of ellipsoidal microparticles (as Janus‐type microparticles). The relevant results of adding this dexMA‐biotin into the PEG/PEGDA phase were shown in Figures S19 and S20. These results reveal that after removal of thiol group and changing the polymer skeleton structure from PEG to dextran, the dexMA‐biotin (DS_biotin_ of 13 %) still selectively compartmentalizes in the interfacial layers adjacent to the PEG/PEGDA phase and migrates along these interfacial layers, but its equilibrium state shifts from only the water (PEG/PEGDA)‐oil interfacial layer to both interfacial layers corresponding to the water (PEG/PEGDA)‐oil interface and the water‐water interface.

The interfacial microcompartmentalization of dexMA‐biotin with lower DS_biotin_ of 6 % was further checked. As shown in Figure 3 b and Figures S21 and S22, when DS_biotin_ decreased to 6 % and the ellipsoidal dexMA particles were crosslinked at fast rate (LAP=8 mg mL^−1^), an interfacial microcompartment of dexMA‐biotin was obtained on the flat side of ellipsoidal particles which could not be achieved before. When the particles were crosslinked at low rate (LAP=2 mg mL^−1^), both the curved and flat sides of ellipsoidal dexMA microparticles were covered with dexMA‐biotin compartment, and even the interior hydrogel was slightly modified with dexMA‐biotin (DS_biotin_ of 6 %). It indicates that with decreasing biotin content, dexMA‐biotin still accumulates in the PEG/PEGDA adjacent interfacial layers, but also diffuses to the dexMA bulk phase and the water (dexMA)‐oil interfacial layer which has never been achieved by using biotin‐PEG‐SH and dexMA‐biotin (DS_biotin_=13 %).

The DS_biotin_ of dexMA‐biotin was continuously decreased to ≈1 %. As shown in Figure 3 c and Figure S23, dexMA‐biotin (DS_biotin_=1 %) was homogeneously modified in the ellipsoidal dexMA microparticles, but compartmentalized at the PEGDA microparticles surface which leads to the patchy crescent‐shaped microparticles. It implies that the distribution of dexMA‐biotin (DS_biotin_=1 %) in the ATPS droplet shifts more from the adjacent interfacial layers to the dexMA bulk phase, but never to the PEGDA bulk phase. Furthermore, biotin groups were completely removed (DS_biotin_=0) and the dye labelled dexMA (dexMA‐FITC) was used as additives in the polymer aqueous systems. As shown in Figure S24, the dexMA‐FITC homogeneously distributed inside the PEGDA microparticles and didn't present interfacial compartmentalization like the biotinylated polymers any more.

So, we conclude that the biotin group on biotinylated polymers acts as a key factor which induces the selective interfacial accumulation (SIA) in the PEG/PEGDA containing aqueous system. The selectivity of this micro‐compartmentalization among different interfacial layers and its equilibrium state can be manipulated by adjusting the polymer skeleton structure and degree of biotin substitution.

## Conclusion

We report a novel interfacial microcompartmentalization approach based on the SIA of biotinylated polymers in 3D phase separation. We surprisingly found that biotinylated polymers accumulated spontaneously and selectively in the PEG/PEGDA adjacent interfacial layers in polymer aqueous systems, which is called selective interfacial accumulation (SIA). By further applying this SIA into the ATPS, the biotinylated polymers selectively accumulated in different interfacial layers and migrated along these interfacial layers. By capturing the interfacial migration kinetics through variation of the crosslinking rates which can be adjusted by the photoinitiator concentration, the migration pathway of these biotinylated polymers has been investigated, and functional interfacial microcompartments of biotinylated polymers have been created during the formation of crescent‐shaped and ellipsoidal hydrogel microparticles in microfluidics. These biotinylated microcompartments could be connected with streptavidin, of which the remaining free biotin binding sites provide a handle for further surface/interface functionalization. By varying polymer skeleton structure and the biotin content (DS_biotin_), the selectivity of biotinylated polymers at different interfacial layers in the ATPS droplet can be further manipulated and various interfacial microcompartments have been obtained.

Altogether, we found the SIA of biotinylated polymers in polymer aqueous systems. By adjusting the polymer compositions, we have been able to kinetically control and capture the spatial organisation in a variety of compartmentalized macroscopic structures, without the need to create barrier layers between compartments. It provides an ingenious and continuous approach to construct functional subcompartments on the surface/interface of soft matter. The existence of this phenomenon not only opens a new avenue for current 3D phase separation approaches towards synthetic compartmentalized systems, but is also highly relevant to the understanding and mimicking of the interlinked membrane and intracellular organisation in living systems.

## Methods

Formation of hydrogel microparticles. All aqueous phases were prepared with demineralized water. The oil phase (hexadecane) was mixed with surfactant (Span 80, 3 % w/w). The three phases were independently injected into the microfluidic device by syringe pumps. The droplets were cross‐linked in chip by UV irradiation (365 nm) and collected from the outlet of the chip. After removal of hexadecane, the microparticles were washed with THF (1 time) and water (5 times), and stored in aqueous solution.

Spherical microparticles. DexMA (25 % w/w) and PEGDA (28.6 % w/w) was separately mixed with biotinylated polymers (4 mg mL^−1^) and LAP (2 mg mL^−1^). The mixture was used as both inner and middle phases, and injected into the oil phase.

Crescent‐shaped microparticles. Dextran (28.6 % w/w, M_W_=20 000) was used as inner phase. PEGDA (28.6 % w/w, M_W_=700) and LAP (8, 5 and 2 mg mL^−1^) were used as middle phase. Biotinylated polymers were added into the dextran/PEGDA phase.

Ellipsoidal microparticles. DexMA (25 % w/w, M_W_=20 000) and LAP (8, 6, 4 and 2 mg mL^−1^) were used as inner phase. PEG (25 % w/w, M_W_=10 000) were used as middle phase. Biotinylated polymers were added into the dexMA/PEG phase.

Streptavidin‐FITC treatment. Aqueous solution of streptavidin‐FITC (8 μg mL^−1^, 2 mL) was prepared and added to a solution of microparticles functionalized with biotinylated polymers as described above. After 2 days, the solution of streptavidin‐FITC was removed and the microparticles were washed with demineralized water (3–5 times) and stored in aqueous solution, as well as protected from light.

## Conflict of interest

The authors declare no conflict of interest.

## Supporting information

As a service to our authors and readers, this journal provides supporting information supplied by the authors. Such materials are peer reviewed and may be re‐organized for online delivery, but are not copy‐edited or typeset. Technical support issues arising from supporting information (other than missing files) should be addressed to the authors.

Supplementary

Supplementary

Supplementary
