# Supplementary material for: Interfacial Microcompartmentalization by Kinetic Control of Selective Interfacial Accumulation
Source: Angew Chem Int Ed Engl. 2020 Oct 25;59(52):23748–54. doi: 10.1002/anie.202009701 (PMC7894335; doi:10.1002/anie.202009701)
Supplement: Supplementary file 1 — Supplementary [file ANIE-59-23748-s001.pdf]

Angewandte Chemie
Eine Zeitschrift der Gesellschaft Deutscher Chemiker

Supporting Information

Interfacial Microcompartmentalization by Kinetic Control of Selective Interfacial Accumulation

*Qian Liu, Zhenyu Yuan, Meng Zhao, Max Huisman, Gido Drewes, Tomasz Piskorz, Serhii Mytnyk, Ger J. M. Koper, Eduardo Mendes, and Jan H. van Esch**

anie_202009701_sm_miscellaneous_information.pdf
anie_202009701_sm_Video_1.avi
anie_202009701_sm_Video_2.avi

Table of Contents

Video 1: Generation of different hydrogel microparticles

Video 2: Hydrogel microparticles with different surface modifications

1. Materials

2. Equipment

3. Fabrication of a microfluidic device

4. Interfacial tension measurement

5. Selective interfacial accumulation (SIA)

5.1 Generation of spherical hydrogel microparticles (**Figure S1**)

5.2 Unmodified hydrogel microparticles (**Figure S2**)

5.3 Interfacial accumulation (**Langmuir-McLean equation**)

6. Kinetic control of the selective interfacial accumulation (SIA)

6.1 Generation of crescent-shaped and ellipsoidal hydrogel microparticles (**Figures S3, S4**)

6.2 Interface identification (**Figure S5**)

6.3 Unmodified crescent-shaped microparticles (**Figure S6**)

6.4 Fractured crescent-shaped and ellipsoidal microparticles (**Figure S7**)

6.5 Crescent-shaped microparticles at different rotation angles (**Figure S8**)

6.6 Microparticles with biotin-PEG-SH being added in dextran/dexMA (**Figures S9, S10, S11, S12, S13**)

6.7 Microparticles with biotin-PEG-SH being added in PEGDA/PEG (**Figure S14**)

7. Manipulation of the selective interfacial accumulation (SIA)

7.1 Synthesis of dexMA-biotin with different DS_{biotin} (**Figures S15, S16**)

7.2 Spherical microparticles modified with dexMA-biotin (**Figures S17, 18, Table S1**)

7.3 Microparticles with dexMA-biotin of DS_{biotin} 13% being added in PEG/PEGDA (**Figure S19**)

7.4 Microparticles at low concentration of dexMA-biotin with DS_{biotin} 13% (**Figure S20**)

7.5 Microparticles with dexMA-biotin of DS_{biotin} 6% (**Figures S21, S22**)

7.8 Microparticles with dexMA-biotin of DS_{biotin} 1% being added in PEG/PEGDA (**Figure S23**)

7.9 Microparticles with dexMA-FITC (**Figure 24**)

Experimental Procedures

1. Materials

Poly(ethylene glycol) diacrylate (PEGDA, M_w=700), poly(ethylene glycol) (PEG, M_w=10000), dextran (M_w=20000), hexadecane, Span 80 and fluorescein isothiocyanate-labelled streptavidin (streptavidin-FITC) were purchased from Sigma-Aldrich (Steinheim, Germany). HS-PEG-biotin (M_w=788) was purchased from Polypure (Oslo, Norway). Methacryloxyethyl thiocarbamoyl rhodamine B was purchased from Polysciences, Inc. The photo initiator: lithium phenyl-2,4,6-trimethylbenzoylphosphinate (LAP) was synthesized as previously described[1]. Rhodamine B-labelled dextran methacrylate (dexMA, M_w=20000, DS=10%) and DexMA-biotin (DS_{biotin}=1%, 6% and 13%, M_w=20000) was synthesized as shown in part 7.

2. Equipment

Syringe Pumps (Harvard Apparatus, Pump 11, Pico Plus Elite). Axio Observer A1 inverted microscope (Zeiss, ×10 air objective) with a Zyla 5.5 sCMOS camera (Andor) at 50 fps. Mercury-arc light source (HXP 120 V,120 W) with a band pass filter 300-400 nm (peak intensity at 365 nm). Drop shape analysis (DSA1 v 1.9, Kruss GmbH). Confocal laser scanning microscopy (CLSM, Zeiss LSM 710, ×10 and ×20 air objectives and a ×40 oil immersion objective). JEOL 6010 Scanning Electron Microscope (SEM).

3. Fabrication of a microfluidic device

The microfluidic device was fabricated by PDMS (Dow Corning, Sylgard 184 elastomer kit) using soft lithography and bonded with two pieces of PDMS after oxygen plasma treatment. The channel height is about 300 µm, width is about 500 µm. The width of nozzles is about 40 µm. The device was connected to individual syringe pumps (Harvard Apparatus, Pump 11, Pico Plus Elite) via a tube (PEEK® 0.5/1.6 mm inner/outer diameter). The volumetric flow rate of inner phase (dextran or dexMA) was 0.02~0.09 µL/min and the volumetric flow rate of middle phase (PEG or PEGDA) was 0.1~0.18 µL/min. The volumetric flow rate of hexadecane was 10~15 µL/min.

4. Interfacial tension measurement

Interfacial tensions of PEGDA and dexMA droplets in oil were measured with a Kruss Easy-Drop. The solution of PEGDA (28.6% w/w, ρ=1.052 g/mL) or dexMA (25% w/w, ρ=1.064 g/mL) with or without PEG-biotin-SH or dexMA-biotin (4 mg/mL) was placed in a syringe. The needle was immersed into a glass cuvette containing 20 mL hexadecane (ρ=0.773 g/mL) with or without surfactant Span 80 (3% w/w). The solution was slowly injected into hexadecane, until a droplet formed. The interfacial tension of this droplet was calculated with the Kruss Drop Analysis software by using the Young-Laplace method.

Results and Discussion

5. Selective interfacial accumulation

5.1 Generation of spherical hydrogel microparticles

Spherical hydrogel microparticles were fabricated by using the same polymer solution in both inner and middle phases in the microfluidic device. For the dexMA particles (see **Figure S1a**), a mixture of dexMA (25% w/w) and biotin-PEG-SH (4 mg/mL) was injected into both the inner and middle channels, hexadecane with Span 80 (3% w/w) was injected into the outer channel. Spherical microdroplets were generated in the outer channel and cross-linked to form the hydrogel by on-chip UV irradiation. After collecting and washing the particles, the diameter of these dexMA particles was in the range from 77 to 98 µm. PEGDA particles (**Figure S1b**) with the diameter from 122 to 145 µm were produced by the same method with PEGDA (28.6% w/w) and biotin-PEG-SH (4 mg/mL).

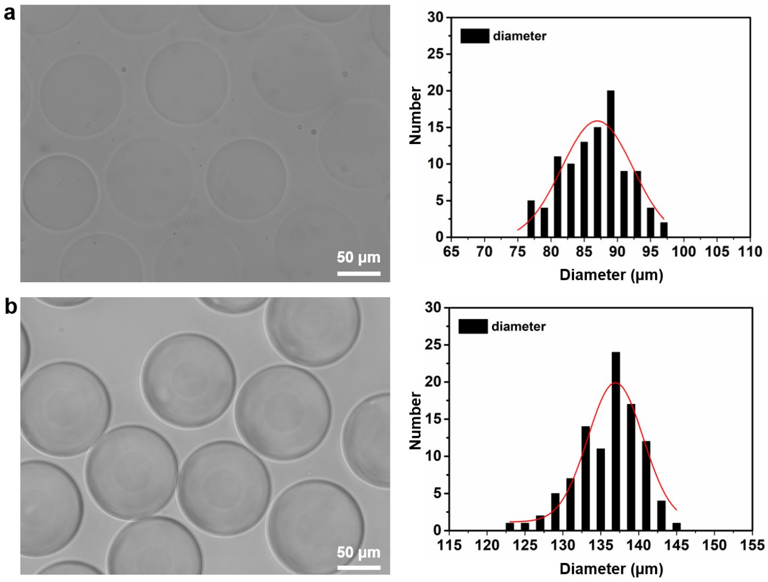

Figure S1. Spherical hydrogel microparticles. Microscope images of dexMA (**a**) and PEGDA (**b**) hydrogel microparticles, and the corresponding diameter distribution.

5.2 Unmodified hydrogel microparticles

Unmodified dexMA microparticles and PEGDA microparticles were reacted with streptavidin-FITC. As shown in **Figure S2**, after reacting the unmodified dexMA and PEGDA microparticles with streptavidin-FITC solution respectively and washing these particles with water (3 times), very weak fluorescent signal was homogeneously present in the particles. It indicates that streptavidin-FITC can diffuse into the dexMA and PEGDA hydrogel network, but can’t bind to the particles in the absence of biotin.

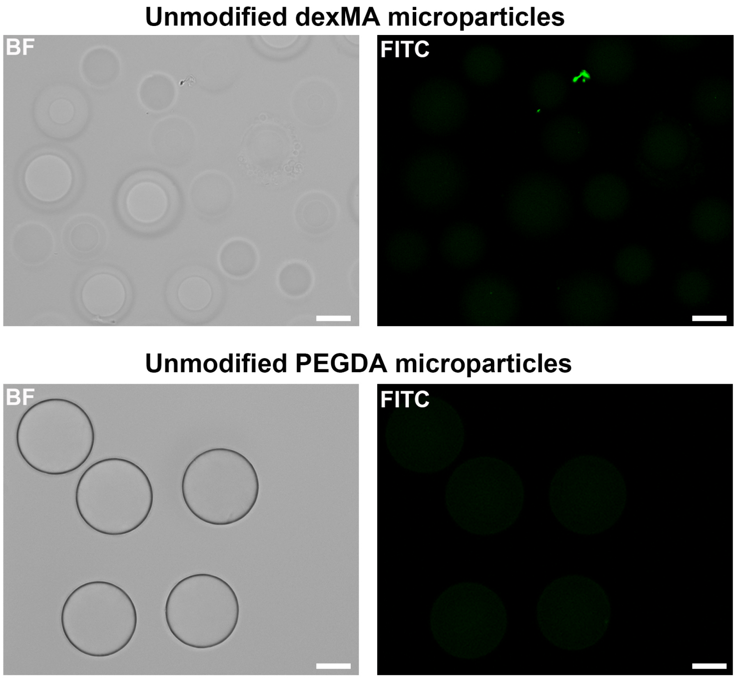

Figure S2. Unmodified spherical hydrogel microparticles. Bright-field (BF) and CLSM images of unmodified dexMA and PEGDA hydrogel microparticles after being reacted with streptavidin-FITC and washed with water (3 times). Scale bar 50 µm.

5.3 Interfacial accumulation

Interfacial accumulation (segregation) refers to the enrichment of atoms, ions, or molecules at the interface in a materials system. Interfacial accumulation of biotin-PEG-SH indicates that the free energy of the whole droplet was lowered when biotin-PEG-SH migrate to the interface or interfacial layer. Relation between the interface concentration of biotin-PEG-SH and the free energy change obeys the **Langmuir-McLean equation** as [*][2]:

$$\frac{X_{\text{interface}}}{1-X_{\text{interface}}}=\frac{X_{\text{bulk}}}{1-X_{\text{bulk}}}.\exp(-\frac{\Delta E}{RT})$$

where $\Delta E = E_{\text{interface}} - E_{\text{bulk}}$ is the energy difference between a biotinylated polymer at interface and in bulk.

The free energy of biotin-PEG-SH consists of two contributions, elastic strain and chemical interaction. Therefore, ΔE can be described as:

$$\Delta E=\left(E_{\text{interface}}^{\text{elastic}}+E_{\text{interface}}^{\text{chemical}}\right)-\left(E_{\text{bulk}}^{\text{elastic}}+E_{\text{bulk}}^{\text{chemical}}\right)=\Delta E_{\text{elastic}}+\Delta E_{\text{chemical}}$$

where E_{elastic} is the elastic strain energy of biotin-PEG-SH, which is directly related to the deformation and distortion of the polymers. E_{chemical} represents the chemical interaction between biotin-PEG-SH and other surrounding molecules, such as water, hexadecane, PEG and dextran. The interaction can be chemical bonding, hydrogen bonding and dispersion force etc.

For E_{elastic}, due to the expulsion between polymers and polymers, or polymers and solvent, there is “depletion region” at the water-water and water-oil interfaces. It provides more “free volume” for the biotin-PEG-SH than the bulk provides. Therefore, the E_{elastic} caused by the molecular deformation can be considerably released at the interface, which makes the interface regions much more energetic preferable than the bulk of the droplets.

For E_{chemical}, the results suggest that the biotin-PEG-SH have unfavourable or favourable interaction with the PEG/PEGDA polymers to push the biotin-PEG-SH to the droplet edge, which leads to a more negative $\Delta E_{\text{chemical}}$. Therefore, we speculate that both elastic strain energy and chemical interaction perhaps play roles to induce the accumulation of biotin-PEG-SH in the interfacial layer, but their preference to the adjacent PEG/PEGDA interfacial layers is mainly attributed to the chemical interaction between the biotinylated polymers and the PEG/PEGDA phase.

6. Kinetic control of the selective interfacial accumulation

6.1 Generation of crescent-shaped and ellipsoidal hydrogel microparticles

Crescent-shaped hydrogel microparticles were fabricated by using a dextran/PEGDA/oil system in the microfluidic device. The morphology of crescent-shaped microparticles was characterized under SEM after freeze-drying. As shown in **Figure S3a**, all the particles have open cavities and uniform morphologies. The size distribution of these microparticles in water was measured under the bright-field microscope. As shown in **Figure S3b**, the diameter of particle was in the range of 130-145 μm and the diameter of the cavity was around 79-100 μm, and the opening size of the cavity slightly decreased to 72-96 μm.

Biotin-PEG-SH (4 mg/mL) was added in the dextran phase, after cross-linking PEGDA phase at 8 mg/mL LAP and removal of the dextran phase, the surface composition of crescent-shaped hydrogel microparticles were checked by SEM/EDS (energy dispersive spectroscopy) analysis. As shown in Figure S3c, N and S elements existed on the particles inner surface. It indicates that the thiol-ene reaction has taken place and the biotin-PEG-SH has been bound to the hydrogel network.

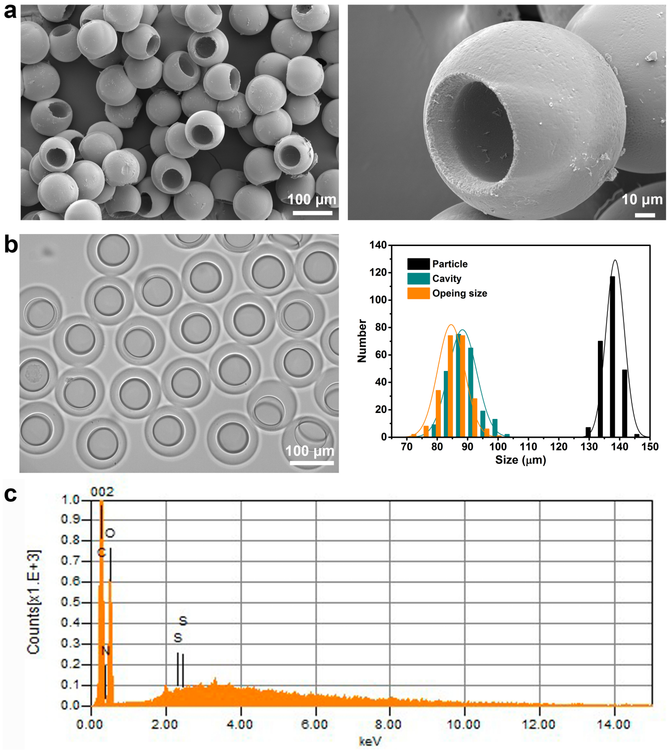

Figure S3. Crescent-shaped hydrogel microparticles. Micrographs of crescent-shaped particles under SEM (**a**). Bright-field microscopy image and the size distribution of crescent-shaped microparticles (**b**). SEM/EDS analysis on the inner surface of crescent-shaped microparticles after cross-linking with biotin-PEG-SH (**c**).

The ellipsoidal hydrogel microparticles were fabricated by using a dexMA/PEG/oil system in the microfluidic device. The size of ellipsoidal particles can be adjusted by varying the volumetric flow rate ratio of dexMA and PEG (F_d: F_p). In the experiment, we obtained ellipsoidal microparticles with two size ranges. As shown in **Figure S4a**, small ellipsoidal microparticles with diameter from 25-70 μm were obtained at volumetric flow rate ratio of 1:5. With the increase of volumetric flow rate ratio to 1:2, the diameter of ellipsoidal microparticles increased to 80-115 μm (**Figure S4b**). The morphology of ellipsoidal microparticles is shown in the inserted microscopy images of particles' side view.

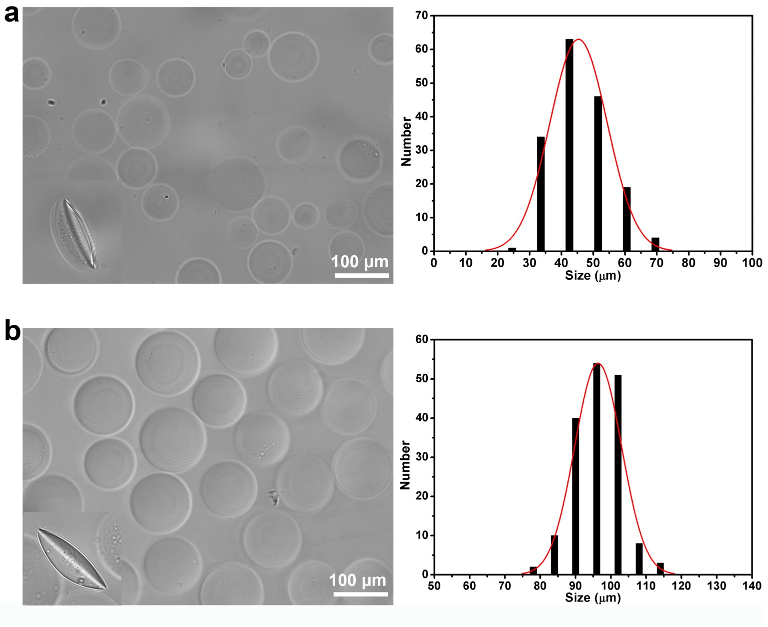

Figure S4. Ellipsoidal hydrogel microparticles. Bright-field microscopy images and diameter distribution of the ellipsoidal hydrogel particles at F_d: F_p of 1:5 (**a**) and 1:2 (**b**). Microscopy images of particles' side view were inserted.

6.2 Interface identification

The ellipsoidal microparticles produced by cross-linking of dexMA are non-centrosymmetric. To identify the morphology of these particles, both PEGDA and dexMA were cross-linked at the same time in the microfluidic device. As shown in **Figure S5**, the microscopy images clearly indicate that the inner surface of the crescent-shaped particles and the curved side of ellipsoidal particles correspond to the water-water interface. The outer surface of the crescent-shaped particles corresponds to the water (PEGDA)-oil interface, and the flat side of the ellipsoidal particles corresponds to the water (dexMA)-oil interface.

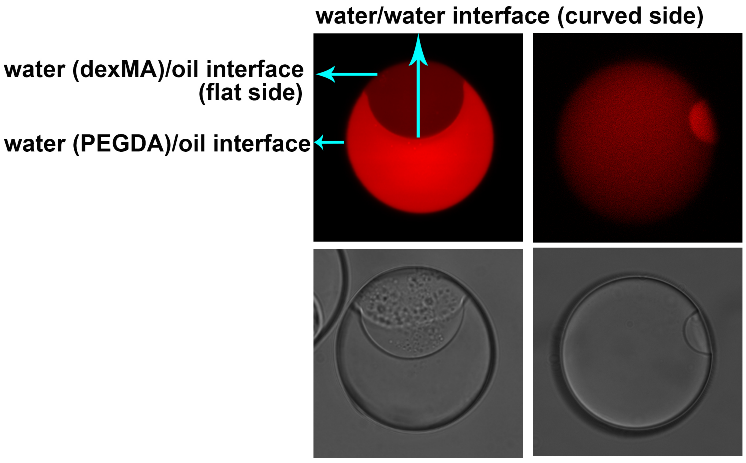

Figure S5. Cross-linking both dexMA and PEDGA. CLSM and bright-field microscopy images of the spherical microparticles by cross-linking both dexMA and PEGDA phases at F_d: F_p of 1:2 (left) and 1:5 (right).

6.3 Unmodified crescent-shaped microparticles

Unmodified crescent-shaped microparticles were reacted with streptavidin-FITC. As shown in **Figure S6**, After reacting with streptavidin-FITC, strong fluorescence signal was observed in the crescent-shaped microparticles before washing these particles with

water. However, after washing with water (3 times), the fluorescence signal obviously decreased. It indicates that, the streptavidin-FITC can diffuse into the PEGDA hydrogel network, but can't stay without the modification of biotinylated polymers.

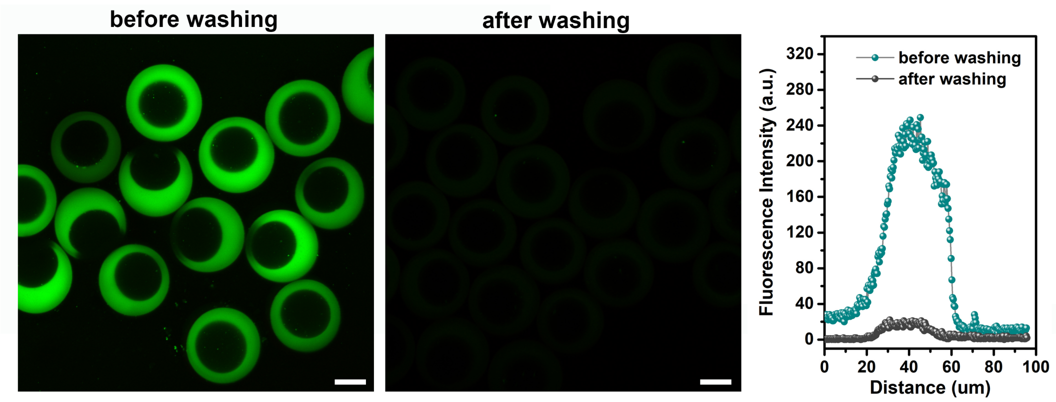

Figure S6. Unmodified crescent-shaped microparticles. CLSM images of the unmodified crescent-shaped PEGDA microparticles after reacting with streptavidin-FITC before and after washing with water, and the corresponding fluorescence intensity. Scale bar 50 μm.

6.4 Fractured crescent-shaped and ellipsoidal microparticles

We fractured the biotin-modified crescent-shaped and ellipsoidal gel particles by exposing them to ultrasound (around 30 min), after which they were reacted with streptavidin-FITC. As shown in **Figure S7**, after washing with water, fluorescence was only observed on the surface of microparticles, while fluorescence was neither observed on the cross-sectional fracture surface, nor inside the hydrogel. This result implies that binding of streptavidin does not lead to a barrier layer at the microparticles surface, while it confirms the absence of biotinylated polymers in the particle interior.

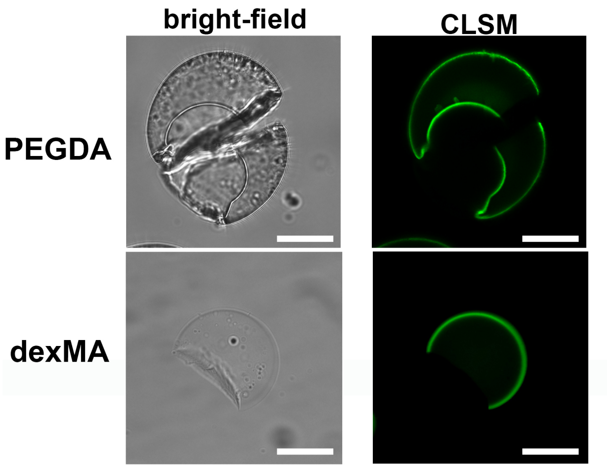

Figure S7. Fractured crescent-shaped PEGDA and ellipsoidal dexMA microparticles. Bright-field microscopy and CLSM images of the fractured PEGDA and dexMA microparticles after reacting with streptavidin-FITC. Scale bar 50 μm.

6.5 Crescent-shaped microparticles at different rotation angles

When biotin-PEG-SH was added into dextran, PEGDA was cross-linked to form crescent-shaped microparticles. As shown in **Figure S8**, the 3D CLSM image of crescent-shaped microparticles at different rotation angles presented that more biotin-PEG-SH molecules were immobilized on the inner surface and the top of outer surface of the crescent-shaped particles, a few showed up on the bottom of the outer surface. So, we speculated that the biotin-PEG-SH did not reach the equilibrium state in the ATPS droplet before

arresting the migration by cross-linking and biotin-PEG-SH molecules' location in the microparticles can be captured at different cross-linking rates.

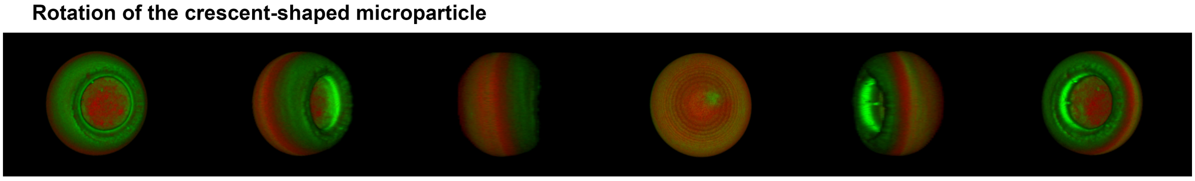

Figure S8. 3D CLSM images of the crescent-shaped microparticles at different rotation angles.

6.6 Microparticles with biotin-PEG-SH being added in dextran/dexMA

Biotin-PEG-SH was compartmentalized at different surfaces of the crescent-shaped microparticles cross-linking with different concentrations of LAP. As shown in **Figure S9**, around 92% particles were modified only on the inner surface with 8 mg/mL of LAP. When the concentration of LAP decreased to 5 mg/mL, both the inner and outer surfaces of 96% particles were covered with the biotin-compartment. When the concentration of LAP decreased to 2 mg/mL, biotin-compartment was only present on the outer surface of the particles. It shows the interfacial compartmentalization of biotin-PEG-SH in the crescent-shaped particles can be well controlled by varying the cross-linking rates which are controlled by different concentrations of LAP.

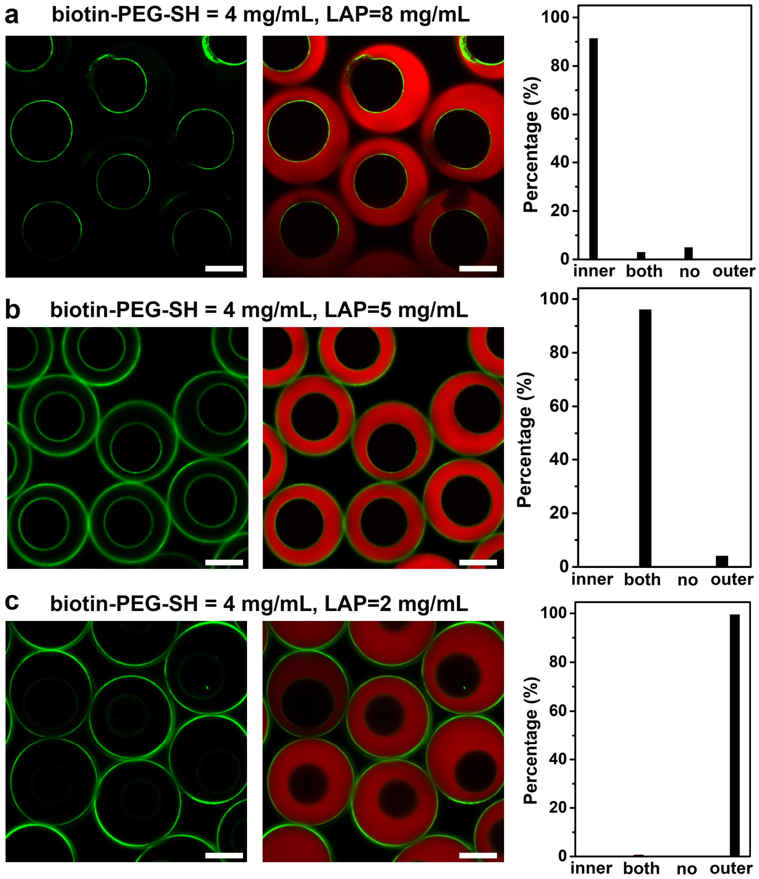

Figure S9. Crescent-shaped microparticles modified with biotin-PEG-SH. When biotin-PEG-SH (4 mg/mL) was added into dextran and PEGDA was cross-linked, CLSM images and statistics of crescent-shaped microparticles modified with biotin-PEG-SH compartment on the inner surface (**a**), both sides (**b**) and the outer surface (**c**). 100 particles for each statistic. Scale bar 50 μm.

The kinetic migration process of biotin-PEG-SH along the interface was also captured by cross-linking the crescent-shaped gel particles in microfluidic channels with different lengths. Biotin-PEG-SH was added into the dextran and the PEGDA was cross-linked at 8 mg/mL of LAP. As shown in **Figure S10**, crescent-shaped particles with only inner surface modification were obtained in the short channel. However, when the particles were cross-linked in the long channel, both the inner and outer surfaces were modified with biotin-PEG-SH and no obvious FITC signal was observed in the gel network. It indicates that the interfacial migration and accumulation strongly depend on the residence time and occur before polymerization, and the biotin-PEG-SH migrates from the water-water interfacial layer to the water (PEGDA)-oil interfacial layer.

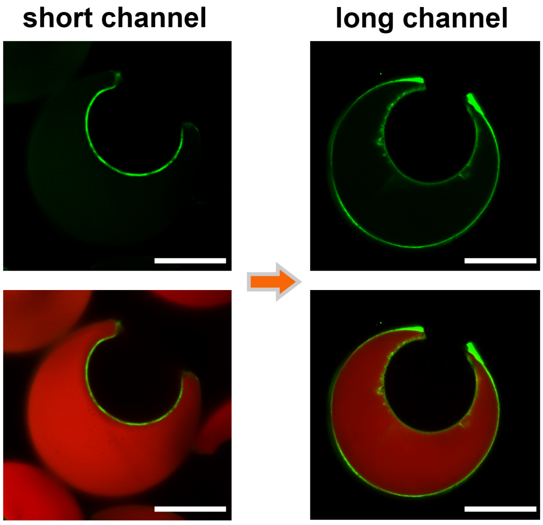

Figure S10. Crescent-shaped microparticles cross-linked in different channels. CLSM images of biotin-modified crescent-shaped particles after being cross-linked in short (left, 3.5 cm) and long (right, 53.5 cm) channels. Scale bar 50 μm.

Influence of the biotin-PEG-SH concentration on the SIA was also investigated. As illustrated in **Figure S11**, when the concentration of biotin-PEG-SH increased to 8 mg/mL, more particles showed biotin-compartments on both sides at high concentration LAP (8 mg/mL). When the particles were cross-linked at low concentration of LAP (2 mg/mL), around 90% particles showed compartment of biotin-PEG-SH on the outer surface. It indicates that biotin-PEG-SH still accumulates in the PEGDA adjacent interfacial layers at high concentration of biotin-PEG-SH.

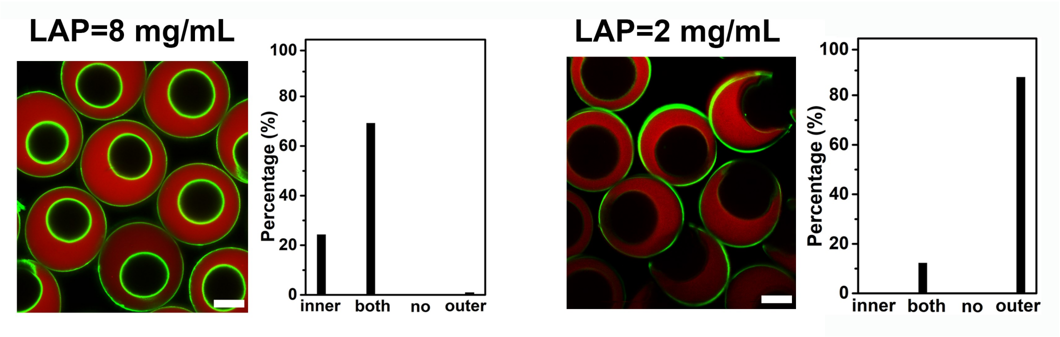

Figure S11. Crescent-shaped microparticles modified with high concentration of biotin-PEG-SH. CLSM images of crescent-shaped PEGDA microparticles (8 mg/mL of biotin-PEG-SH added in dextran) cross-linked at 8 (left) and 2 (right) mg/mL of LAP, and the corresponding statistics of the biotin-compartment distribution (100 microparticles for each condition). Scale bar 50 μm.

Cavity size of the crescent-shaped microparticles can be adjusted by varying the volumetric flow rate ratio of dextran and PEGDA (F_d: F_p). As shown in the **Figure S12a**, with the decrease of F_d: F_p, cavity size of the crescent-shaped microparticles decreased and all the inner surfaces were modified with biotin-PEG-SH at 8 mg/mL LAP. When the concentration of dextran was decreased to 23% (w/w) and biotin-PEG-SH was added into the dextran, 90% of the crescent-shaped particles were asymmetrically modified with biotin on the inner surface at LAP 8 mg/mL (see **Figure S12b**). It indicates that the volumetric flow rates and concentrations of dextran have no obvious influence on the selective interfacial accumulation of the biotin-PEG-SH.

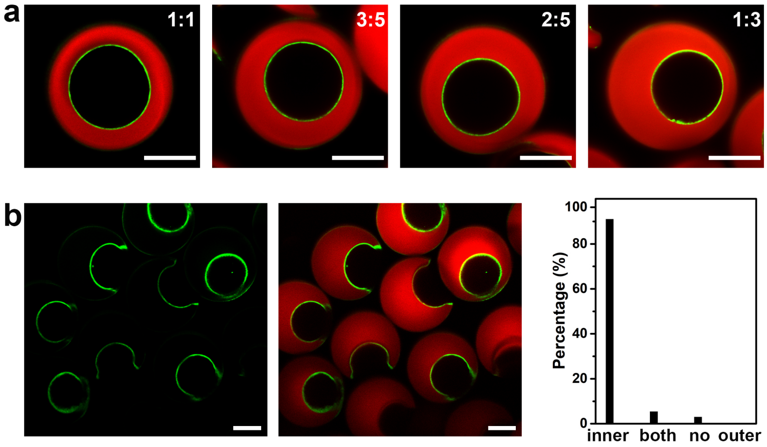

Figure S12. Crescent-shaped microparticles created at different conditions. CLSM images of crescent-shaped microparticles with inner surface modification created by different F_d: F_p (**a**) and concentration of dextran (**b**). The statistics of biotin-compartment distribution was from 100 particles. Scale bar 50 µm.

Biotin-PEG-SH was compartmentalized in the ellipsoidal microparticles by adding it in dexMA and cross-linked dexMA at different concentrations of LAP. As shown in **Figure S13**, when the dexMA was cross-linked fast, around 92% ellipsoidal particles were modified with biotin-compartment on both the curved side and in bulk. However, with the decrease of LAP concentration to 6 mg/mL, 95% of the ellipsoidal particles were covered by biotin-compartment only on their curved sides, as shells. When the concentration of LAP decreased to 4 mg/mL, 18% of the ellipsoidal particles were unmodified, and 82% of the particles were still modified with biotin-compartment on their curved sides, but as rings. When the concentration of LAP decreased more (2 mg/mL), 100% of the particles were without modification.

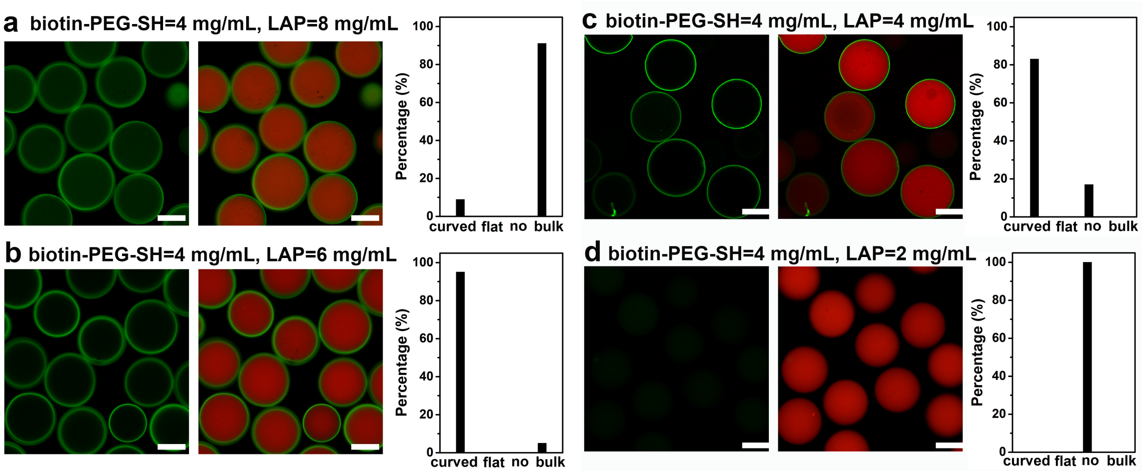

Figure S13. Ellipsoidal microparticles modified with biotin-PEG-SH. When biotin-PEG-SH (4 mg/mL) was added into dexMA and dexMA was cross-linked, CLSM images and statistics of biotin-modified ellipsoidal microparticles cross-linked at 8 mg/mL (**a**), 6 mg/mL (**b**), 4 mg/mL (**c**) and 2 mg/mL (**d**) of LAP. 100 particles for each statistic. Scale bar 50 μm.

6.7 Microparticles with biotin-PEG-SH being added in PEGDA/PEG

When the biotin-PEG-SH was added into PEGDA and PEG to form the crescent-shaped microparticles and ellipsoidal microparticles, various types of interfacial compartmentalization on the microparticles were obtained. As shown in **Figure S14**, at 8 mg/mL of LAP, 93% of the crescent-shaped microparticles were modified with biotin-compartment on both inner and outer surfaces, and all ellipsoidal microparticles were modified on the curved side with thin layers of biotin-PEG-SH. When the concentration of LAP decreased to 2 mg/mL, biotin-compartment was present on the outer surface of 95% of the crescent-shaped particles and no modification was observed on the ellipsoidal microparticles.

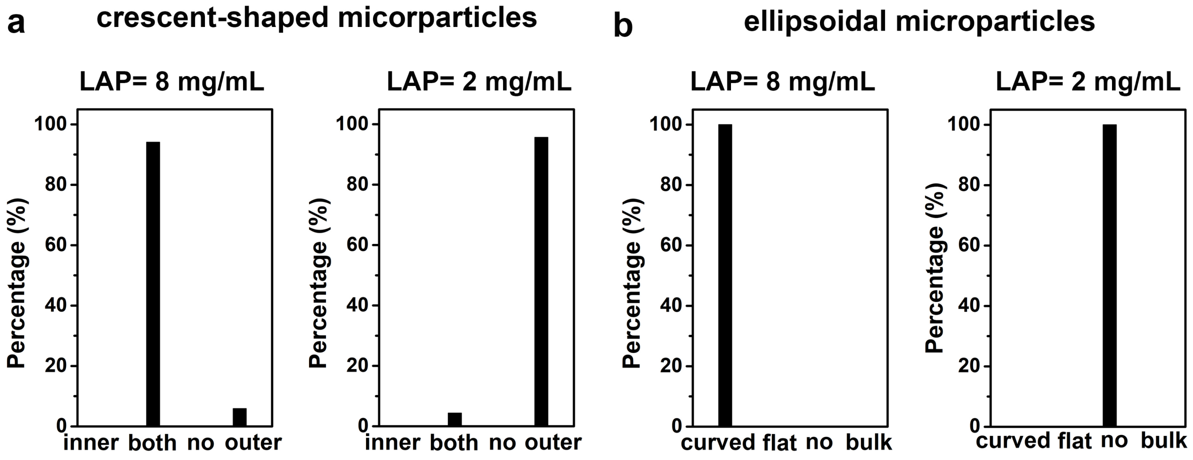

Figure S14. Statistics of biotin-compartment distribution on the crescent-shaped (**a**) and ellipsoidal (**b**) microparticles at different concentrations of LAP when biotin-PEG-SH was added in PEGDA /PEG. 100 particles for each statistic.

7. Manipulation of the selective interfacial accumulation

7.1 Synthesis of dexMA-biotin with different DS_{biotin}

DexMA was synthesized by dextran (M_w=20000) and glycidyl methacrylate (GMA) as described in literature[3]. Three types of dexMA with different degrees of methacrylate (MA) substitution (DS_{MA}) of 15%, 10% and 5% have been checked. DexMA with DS_{MA} 15% presented poor water solubility and high viscosity leading to unstable droplet generation. DexMA with low DS_{MA} of 5% showed better water solubility, but low reactivity which causes unsuccessful cross-linking in short UV irradiation time (2-3 seconds). DexMA with the DS_{MA} of 10% presented optimal balance between the water solubility and reaction reactivity. So dexMA with DS_{MA} of 10% (see **Figure S15**) was used in our experiment. The solution of dexMA (1 g, 4 mmol) and biotin (1.5 g, 6 mmol) in DMSO (25 mL) was added dropwise into a DMSO solution (5 mL) of 1,3-dicyclohexylcarbodiimide (DCC) (2.5 g, 8 mmol) and 4-(N,N-dimethylamino)pyridine (DMAP) (0.4 g, 2 mmol). The mixture was stirred under nitrogen atmosphere at room temperature for 2 days. The solution was dropped into ethanol and white precipitate was formed. The precipitate was placed into dialysis bags (M_w 500) and dialyzed for 2 weeks against demineralized water. After freeze-drying, a white fluffy product was obtained. The DS_{biotin} on dexMA-biotin can be adjusted by using different ratios of dexMA and biotin. As shown in **Figure S16**, dexMA-biotin with DS_{biotin} of 13%, 6%, and 1% was synthesized in these experiments.

Interfacial tension of the dexMA and PEGDA after adding dexMA-biotin with DS_{biotin} of 13%, 6%, and 1% were measured. As shown in **Table S1**, no significant reduction of the interfacial tension was observed after adding dexMA-biotin with different DS_{biotin} of 13%, 6% and 1% in both PEGDA and dexMA, neither with nor without surfactant (Span 80) in oil. It indicates that the interfacial accumulation is not driven by the amphiphilic character of biotinylated polymers.

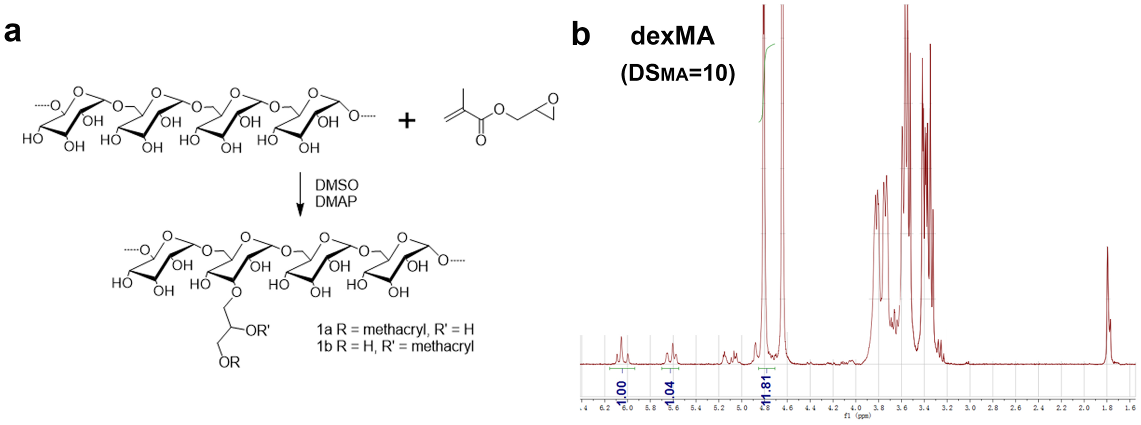

Figure S15. Chemical reaction of the synthesis of dexMA (**a**) and the corresponding ^{1}H NMR spectra (**b**) in D_2O.

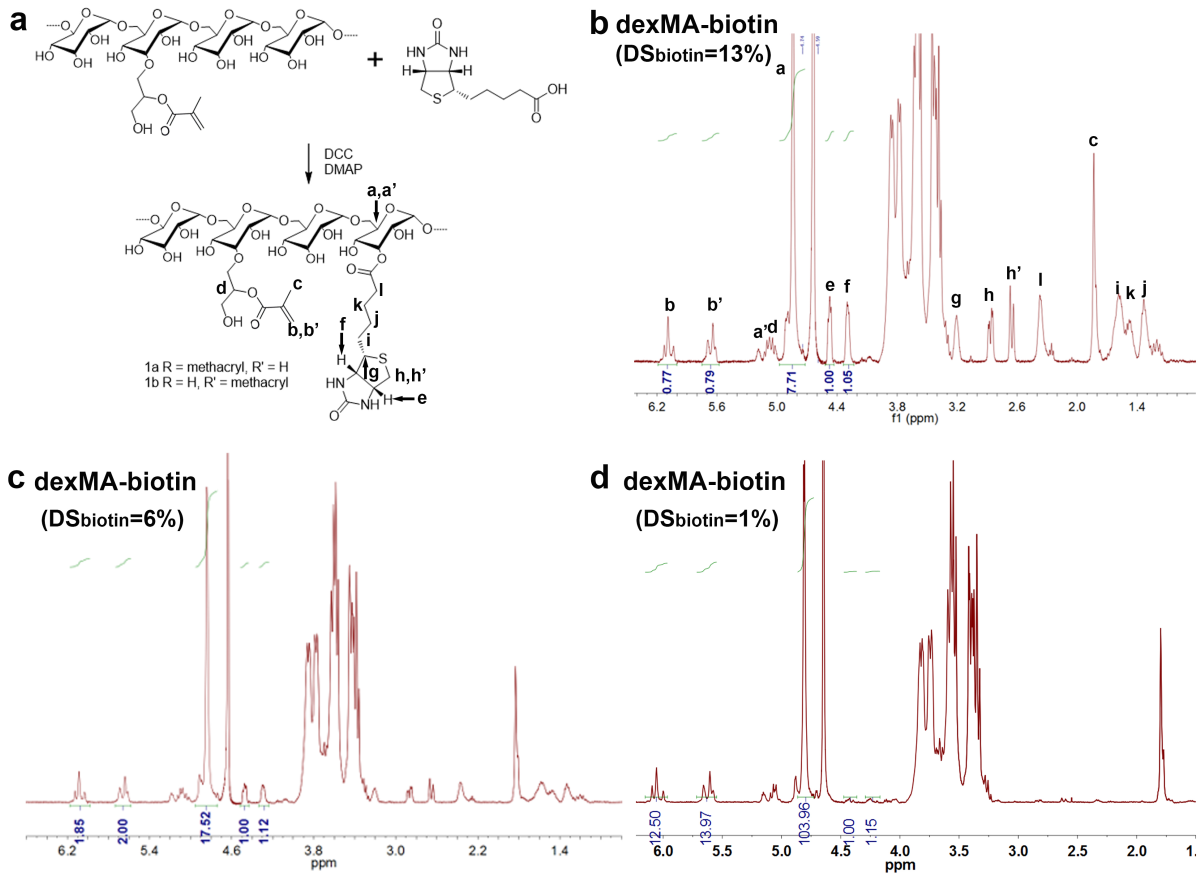

Figure S16. Chemical reaction of the synthesis of dexMA-biotin (**a**) and ^{1}H NMR of dexMA-biotin with DS_{biotin} of 13% (**b**), 6% (**c**) and 1% (**d**) in D_2O.

With Span80	DS_{biotin} 13%	DS_{biotin} 6%	DS_{biotin} 1%	no
**PEGDA**	2.1±0.1	2.0±0.1	2.1±0.1	2.2±0.1
**dexMA**	3.3±0.1	3.4±0.2	3.3±0.1	3.1±0.3

Without Span80	DS_{biotin} 13%	DS_{biotin} 6%	DS_{biotin} 1%	no
**PEGDA**	13.5±0.3	12.7±0.1	13.1±0.2	13.7±0.1
**dexMA**	17.0±0.5	18.6±0.6	18.8±0.6	20.3±0.2

Table S1. Interfacial tension (mN/m) of PEGDA (28.6% w/w) and dexMA (25% w/w) droplets in oil phase in the absence and presence of dexMA-biotin with different DS_{biotin} of 13%, 6% and 1%. Hexadecane with (left) and without (right) Span 80 were used as the oil phase.

7.2 Spherical microparticles modified with dexMA-biotin

Spherical dexMA and PEGDA microparticles were separately modified with dexMA-biotin (DS_{biotin}=13%). As shown in **Figure S17a and S17b**, after reacting with streptavidin-FITC, the dexMA microparticles presented a homogeneous fluorescent signal. However, the PEGDA microparticles only presented green fluorescence on their surface. It implies that dexMA-biotin selectively accumulates in the interfacial layer of PEGDA microparticles.

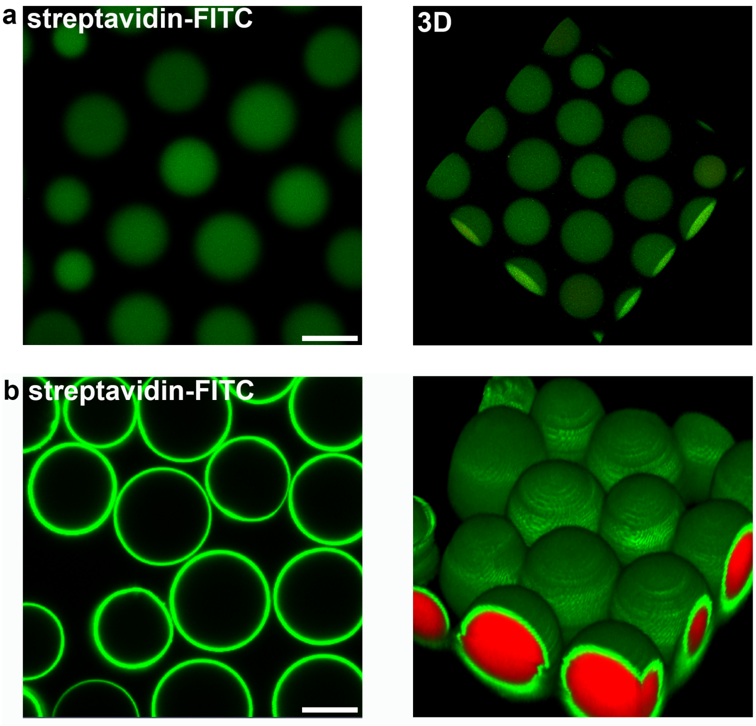

Figure S17. Spherical microparticles modified with dexMA-biotin (DS_{biotin}=13%). CLSM images of dexMA **(a)** and PEGDA **(b)** particles. The left images were scanned with FITC channel, and 3D images were merged by FITC and rhodamine B channels. DexMA and PEGDA particles were labelled with rhodamine B. Scale bar 100 μm.

Spherical microparticles were also obtained by cross-linking both dexMA and PEGDA. As shown in **Figure S18**, dexMA-biotin (DS_{biotin}=13%) was added into the dexMA. After cross-linking dexMA and PEGDA, as well as reacting with streptavidin-FITC, only the surface which next to the water (PEGDA)-oil interface was covered with dexMA-biotin compartment due to its selective interfacial accumulation.

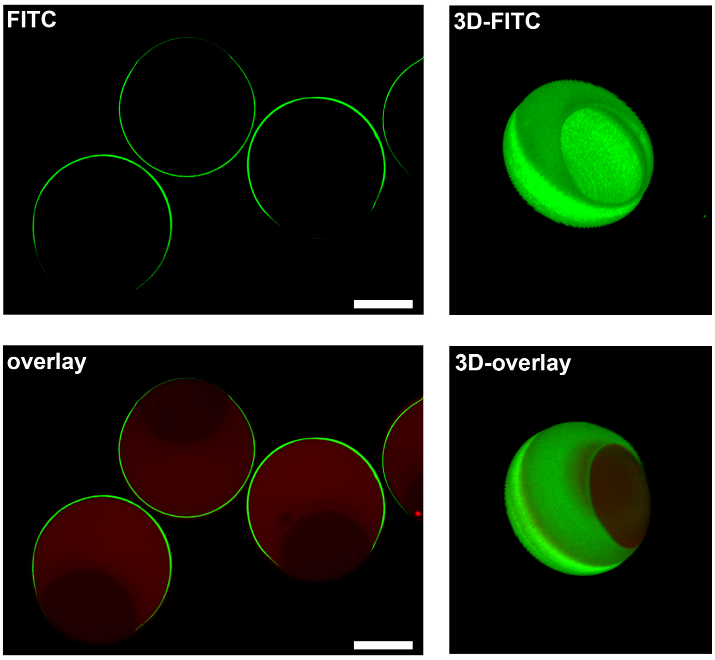

Figure S18. Spherical microparticles produced by cross-linking dexMA and PEGDA. DexMA-biotin (DS_{biotin}=13%, 4mg/mL) was added into the dexMA, both dexMA and PEGDA were labelled by rhodamine B (red) and cross-linked with 2 mg/mL of LAP (in both phases). Scale bar 50 μm.

7.3 Microparticles with dexMA-biotin of DS_{biotin} 13% being added in PEG/PEGDA

Various types of interfacial compartmentalization on the hydrogel microparticles have been achieved by adding dexMA-biotin (DS_{biotin}=13%) in PEG/PEGDA. As shown in **Figure S19**, for the crescent-shaped hydrogel microparticles which were cross-linked at 8 and 2 mg/mL LAP, both inner and outer surfaces of the particles were modified with compartment of dexMA-biotin. For the ellipsoidal microparticles which were cross-linked fast (LAP=8 mg/mL), the curved side of most ellipsoidal microparticles was modified with a small amount of dexMA-biotin and 18% of the particles were unmodified, due to the uncompleted migration of dexMA-biotin from PEG to the interfacial layer under the condition of fast cross-linking. When dexMA-biotin had enough time to diffuse to the interfacial layer during slow cross-linking (LAP=2 mg/mL), 100% of the ellipsoidal particles were asymmetrically modified with a uniform shell of dexMA-biotin on the curved side, like Janus-type microparticles. It indicates that dexMA-biotin (DS_{biotin}=13%) can accumulate in both interfacial layers which correspond to the water (PEGDA)-oil and water-water interfaces.

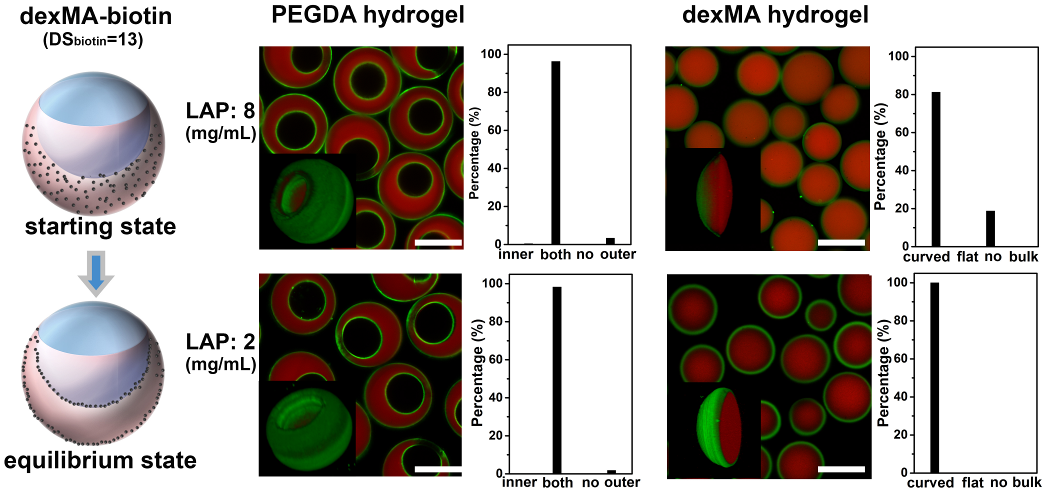

Figure S19. Microparticles modified with dexMA-biotin (DS_{biotin}=13%). CLSM images of biotin-modified crescent-shaped and ellipsoidal microparticles, as well as the corresponding statistics of biotin-compartment distribution under the condition of 8 mg/mL (up) and 2 mg/mL (down) of LAP. 100 particles for each statistic. Scale bar 100 μm.

7.4 Microparticles at low concentration of dexMA-biotin with DS_{biotin} 13%

Low concentration of dexMA-biotin (2 mg/mL) was separately added in PEG and dexMA to form the ellipsoidal microparticles. As shown in **Figure S20**, dexMA-biotin compartment was still only present on the curved side of ellipsoidal particles, but not in a uniform shell anymore (compared with the particles made at 4 mg/mL dexMA-biotin). The top part of ellipsoidal particles has a weaker intensity than in other parts, because of the reduction of dexMA-biotin polymers. It indicates that the concentration of biotinylated polymers has no obvious influence on their equilibrium state in the ATPS droplet.

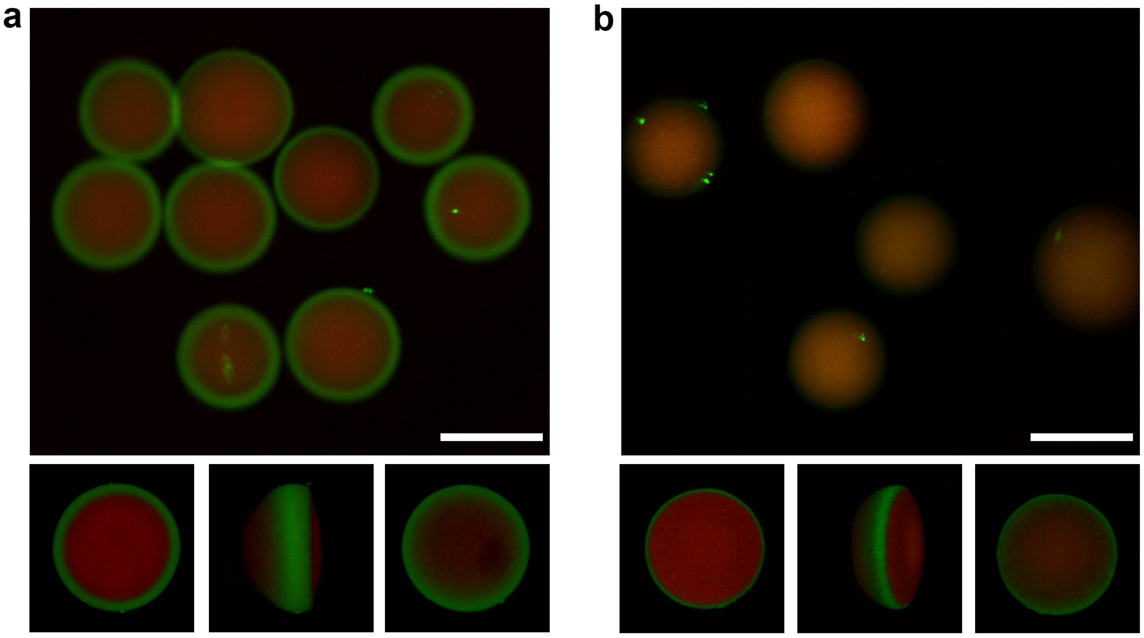

Figure S20. Ellipsoidal microparticles modified with 2 mg/mL of dexMA-biotin. CLSM and images of ellipsoidal particles modified with dexMA (DS_{biotin}=13%) being added in PEG (**a**) and dexMA (**b**). The concentration of LAP was 2 mg/mL. 3D images showed different views of the particles from front (left), side (middle) and back (right). Scale bar 100 μm.

7.5 Microparticles with dexMA-biotin of DS_{biotin} 6%

Different kinds of interfacial compartmentalization on the crescent-shaped and ellipsoidal microparticles have been achieved with the decrease of DS_{biotin} of dexMA-biotin from 13% to 6%. As shown in **Figure S21a**, crescent-shaped particles with biotin-compartment on both inner and outer surfaces were obtained by adding dexMA-biotin (DS_{biotin}=6%) into the dextran. When the particles were cross-linked fast, 34% particles showed inner surface compartmentalization and 67% particles presented both sides compartmentalization, due to the insufficient time for the migration of dexMA-biotin. When the cross-linking rate decreased under the condition of 2 mg/mL of LAP, most gel particles were modified with dexMA-biotin compartment on both inner and outer surfaces (see **Figure S21b**).

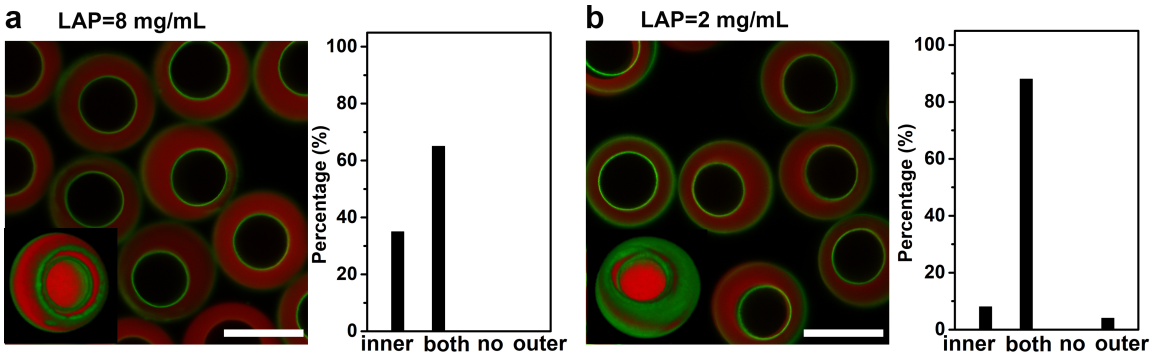

Figure S21. Crescent-shaped microparticles modified with dexMA-biotin (DS_{biotin}=6%) being added in dextran. CLSM images and the statistics of biotin-compartment distribution of the crescent-shaped particles created by adding dexMA-biotin (4 mg/mL) in dextran and cross-linking PEGDA at 8 (**a**) and 2 (**b**) mg/mL of LAP. 100 particles for each statistic. Scale bar 100 μm.

Interfacial compartmentalization in the crescent-shaped and ellipsoidal microparticles created by adding dexMA-biotin (DS_{biotin}=6%) in PEG/PEGDA was also checked. DexMA-biotin was mostly compartmentalized on both curved and flat sides of the ellipsoidal microparticles at high and low concentration of LAP (see **Figure S22a** and **S22c**). Similar results were obtained for the crescent-shaped microparticles with modification of dexMA-biotin compartment on both surfaces (**Figure S22b** and **S22d**). These results indicate that dexMA-biotin (DS_{biotin}=6%) can accumulate in the interfacial layers corresponding to the water-water, water (dexMA)-oil and water (PEGDA)-oil interfaces, and its equilibrium state can be adjusted by varying the DS_{biotin}.

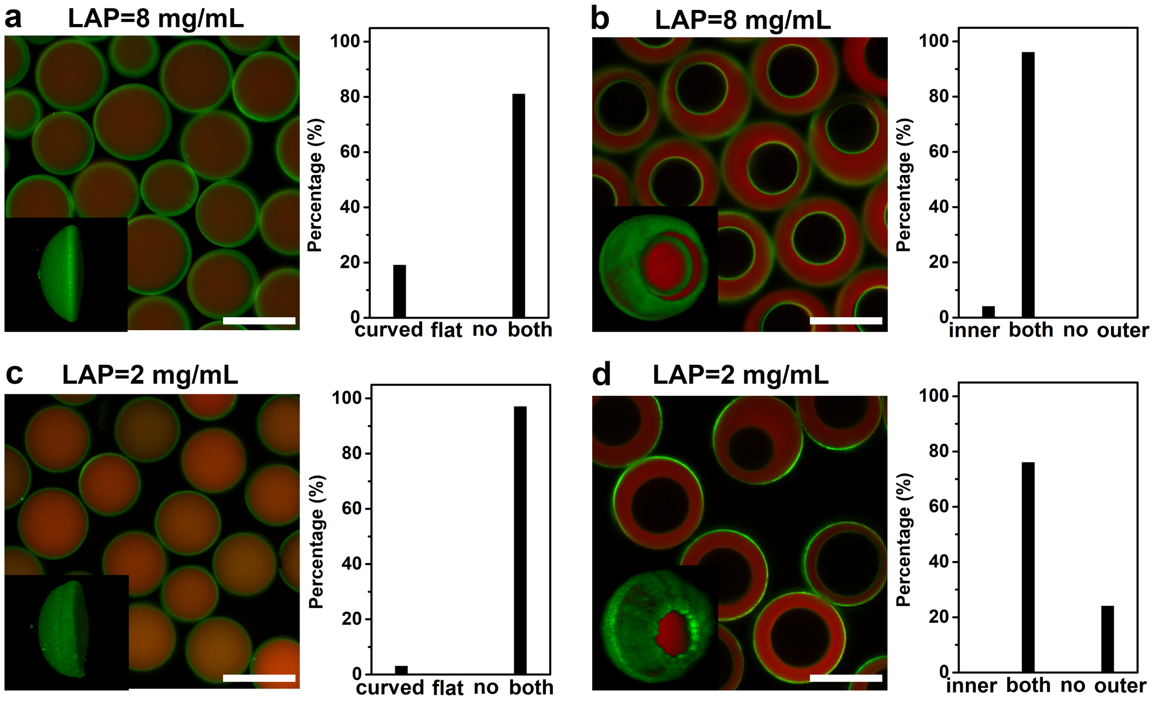

Figure S22. Microparticles modified with dexMA-biotin (DS_{biotin}=6%) being added in PEG/PEGDA. DexMA-biotin (4 mg/mL) was added in PEG/PEGDA. At 8 mg/mL of LAP, CLSM images and the statistics of biotin-compartment distribution of ellipsoidal (**a**) and crescent-shaped (**b**) microparticles. At 2 mg/mL of LAP, CLSM images and the statistics of ellipsoidal (**c**) and crescent-shaped (**d**) microparticles. 100 particles for each statistic. Scale bar 100 µm.

7.6 Microparticles with dexMA-biotin of DS_{biotin} 1% being added in PEG/PEGDA

When DS_{biotin} decreased to 1%, interfacial compartmentalization in the ellipsoidal and crescent-shaped microparticles with dexMA-biotin being added in PEG/PEGDA was checked. As shown in **Figure S23a**, dexMA-biotin (DS_{biotin}=1%) was added into PEG and the dexMA was cross-linked. DexMA-biotin was present on both surfaces of the particles and even inside the dexMA hydrogel. When dexMA-biotin (DS_{biotin}=1%) was added into PEGDA and PEGDA was cross-linked (**Figure S23b**), more than 90% crescent-shaped particles were modified with patches of dexMA-biotin compartment on their inner and outer surfaces. It indicates that the equilibrium state of dexMA-biotin shifts to the dextran/dexMA bulk solution with the decrease of DS_{biotin}.

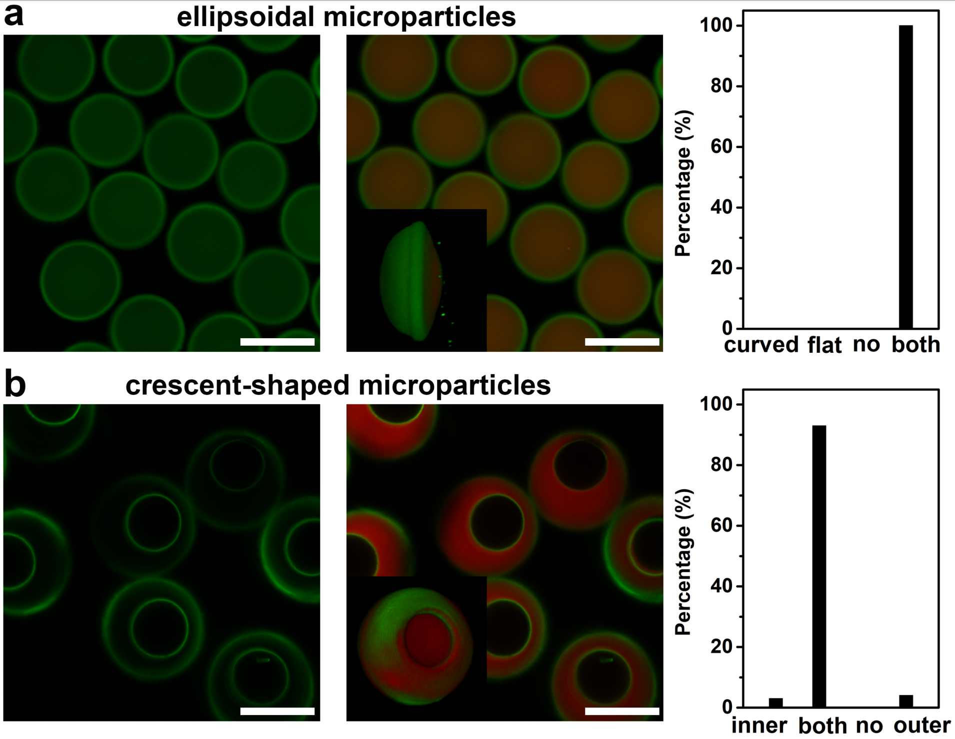

Figure S23. Microparticles modified with dexMA-biotin (DS_{biotin}=1%). At 2 mg/mL of LAP and 4 mg/mL of dexMA-biotin (in PEG/PEGDA), CLSM images and the statistics of biotin-compartment distribution in ellipsoidal particles (**a**) and crescent-shaped particles (**b**). Scale bar 100 µm.

7.7 Microparticles with dexMA-FITC

Biotin groups were completely removed (DS_{biotin}=0) and dye labelled dexMA (dexMA-FITC) was used as an additive in the ATPS. Instead of dexMA-biotin, dexMA-FITC was added in the PEGDA phase to form spherical and crescent-shaped microparticles, respectively. As shown in **Figure S24a**, dexMA-FITC remained homogeneously distributed inside the spherical PEGDA microparticles when the concentration of dexMA-FITC was 4 mg/mL (similar to dexMA-biotin). This low concentration of dexMA-FITC could not cause normal phase separation with PEGDA. When the concentration of dexMA-FITC increased to 28 mg/mL, weak phase separation was observed in the crescent-shaped microparticles by directly adding dexMA-FITC in PEGDA (see **Figure S24b**). It's worth to mention that, the concentration and DS_{dye} of dye labelled dexMA has an important influence on the phase separation[4]. The lower concentration and DS_{dye} of dye labelled dexMA, the more homogeneous modification can be achieved. These results show that phase separation could be achieved by other polymers at high concentration, but without interfacial selectivity. Meanwhile, the biotin group is highly relevant to the selective interfacial accumulation.

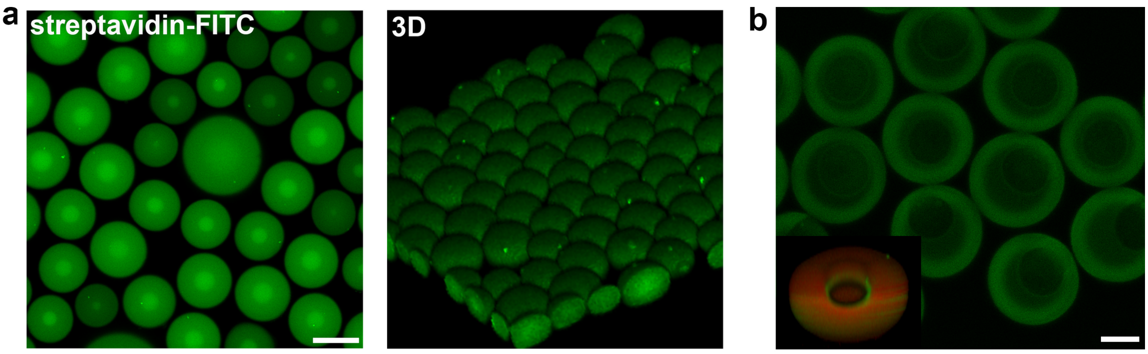

Figure S24. Spherical (**a**) and crescent-shaped (**b**) microparticles cross-linked with dexMA-FITC being added in PEGDA. DexMA-FITC was the mixture of dexMA-FITC and dexMA with a ratio of 1:100 (w/w). Scale bar 50 μm.

References

[1] Tetsuro, M., Worfram, S., *Makromol. Chem.* **2006**, *192*, 2307-2315.
[2] E. D. Hondros, M. P. Seah, *Metall. Mater. Trans. A* **1977**, *8*, 1363-1371.
[3] W. N. E. van Dijk-Wolthuis, O. Franssen, H. Talsma, M. J. van Steenbergen, J J. Kettenes-van den Bosch, W. E. Henninkt, *Macromolecules* **1995**, *28*, 6317-6322.
[4] R. H. Tromp, M. Vis, B. Erné, E. Blokhuis, *J. Phys.: Condens. Matter* **2014**, *26*, 464101.

Author Contributions

Q.L. and J.H.v.E. designed experiments; Q.L. found the phenomenon of selective interfacial accumulation and performed experiments with PEG-biotin-SH; Z.Y. performed experiments with dexMA-biotin (DS_{biotin}=13%); M.Z. performed interfacial tension measurements and exploration of selective interfacial accumulation; M.H. performed experiments with dexMA-biotin (DS_{biotin}=6% and 1%); G.D. performed the experiments with dye labeled dexMA; T.P., G.J.M.K., E.M. helped with data analysis and development of the theory; S.M. designed the microfluidic device and helped with the data analysis; Q.L. and J.H.v.E. analyzed data, wrote the manuscript and supervised the project. All authors edited and approved the final manuscript.
